# The immune microenvironment of HPV-positive and HPV-negative oropharyngeal squamous cell carcinoma: a multiparametric quantitative and spatial analysis unveils a rationale to target treatment-naïve tumors with immune checkpoint inhibitors

**DOI:** 10.1186/s13046-022-02481-4

**Published:** 2022-09-20

**Authors:** Anna Tosi, Beatrice Parisatto, Anna Menegaldo, Giacomo Spinato, Maria Guido, Annarosa Del Mistro, Rossana Bussani, Fabrizio Zanconati, Margherita Tofanelli, Giancarlo Tirelli, Paolo Boscolo-Rizzo, Antonio Rosato

**Affiliations:** 1 Immunology and Molecular Oncology Diagnostics, Veneto Institute of Oncology IOV-IRCCS, Via Gattamelata 64, 35128 Padova, Italy; 2 Department of Surgery, Oncology and Gastroenterology, University of Padova, Padova, Italy; 3 Department of Neurosciences, Section of Otolaryngology, University of Padova, Treviso, Italy; 4 Department of Medicine-DIMED, Section of Pathology, University of Padova, Treviso, Italy; 5 Department of Medical, Surgical and Health Sciences, Section of Pathology, University of Trieste, Trieste, Italy; 6 Department of Medical, Surgical and Health Sciences, Section of Otolaryngology, University of Trieste, Trieste, Italy

**Keywords:** Head and neck squamous cell carcinoma, Human papillomavirus, Tumor microenvironment, Immunotherapy, Multiplex immunofluorescence, Oropharyngeal carcinoma, Gene expression profile, Sex

## Abstract

**Background:**

Immune checkpoint inhibitors (ICI) are approved for treatment of recurrent or metastatic oropharyngeal head and neck squamous cell carcinoma in the first- and second-line settings. However, only 15–20% of patients benefit from this treatment, a feature increasingly ascribed to the peculiar characteristics of the tumor immune microenvironment (TIME).

**Methods:**

Immune-related gene expression profiling (GEP) and multiplex immunofluorescence (mIF) including spatial proximity analysis, were used to characterize the TIME of 39 treatment-naïve oropharyngeal squamous cell carcinomas (OPSCC) and the corresponding lymph node metastases. GEP and mIF results were correlated with disease-free survival (DFS).

HPV-positive tumors disclosed a stronger activation of several immune signalling pathways, as well as a higher expression of genes related to total tumor-infiltrating lymphocytes, CD8 T cells, cytotoxic cells and exhausted CD8 cells, than HPV-negative patients. Accordingly, mIF revealed that HPV-positive lesions were heavily infiltrated as compared to HPV-negative counterparts, with a higher density of T cells and checkpoint molecules. CD8+ T cells appeared in closer proximity to tumor cells, CD163+ macrophages and FoxP3+ cells in HPV-positive primary tumors, and related metastases. In HPV-positive lesions, PD-L1 expression was increased as compared to HPV-negative samples, and PD-L1+ tumor cells and macrophages were closer to PD-1+ cytotoxic T lymphocytes. Considering the whole cohort, a positive correlation was observed between DFS and higher levels of activating immune signatures and T cell responses, higher density of PD-1+ T cells and their closer proximity to tumor cells or PD-L1+ macrophages. HPV-positive patients with higher infiltration of T cells and macrophages had a longer DFS, while CD163+ macrophages had a negative role in prognosis of HPV-negative patients.

**Conclusions:**

Our results suggest that checkpoint expression may reflect an ongoing antitumor immune response. Thus, these observations provide the rationale for the incorporation of ICI in the loco-regional therapy strategies for patients with heavily infiltrated treatment-naïve OPSCC, and for the combination of ICI with tumor-specific T cell response inducers or TAM modulators for the “cold” OPSCC counterparts.

**Supplementary Information:**

The online version contains supplementary material available at 10.1186/s13046-022-02481-4.

## Background

Among head and neck squamous cell carcinomas (HNSCC), oropharyngeal squamous cell carcinomas (OPSCC) comprise cancers of the palatine tonsils, base of tongue, soft palate and posterior pharyngeal wall [1], and are usually associated with tobacco and alcohol consumption [2]. However, carcinogenic high-risk human papillomavirus (HPV) infection has emerged as an important risk factor causing an increase in the incidence of OPSCC over the past 20 years, and being responsible for 71 and 52% of all OPSCC in the USA and UK, respectively [3]. These tumors mainly arise from the reticulated epithelium lining the crypts of the palatine tonsils and the base of tongue which are the preferential target of HPV transforming infection [4]. HPV-positive cancers represent a separate entity characterized by a distinct genetic profile, a platform-independent better response to treatment and a higher chemo- and radio sensitivity, which result in a significantly longer overall survival compared with HPV-negative tumors [5, 6]. The immunological response against viral antigens may contribute to the more favorable clinical course, as the HPV-positive tumor immune microenvironment (TIME) is more enriched than the HPV-negative counterpart [7, 8]. In this regard, the presence of cytotoxic T lymphocytes (CTL) specifically directed against HPV16 E6 and E7 proteins has been reported in cervical carcinoma and in OPSCC patients, and correlated with an improved survival [9]. However, the high tumor HPV-antigen load results in a high expression of immune checkpoint genes on tumor cells (e.g., indoleamine 2, 3-dioxygenase 1, IDO-1), and in dysfunction of HPV-specific CTL [10]. In addition, the role of tissue-resident memory (T_RM_) CD8+ T cells co-expressing the CD103 marker has recently emerged as a favorable prognostic indicator in many cancer types, included HNSCC [11–14].

Immune checkpoint inhibitors (ICI) targeting the PD-1/PD-L1 pathway have been approved for recurrent and metastatic HNSCC patients in the first- and second-line settings [15–17]. However, the role of ICI in OPSCC is still controversial, as only a small proportion of patients benefit from anti-PD-1 monotherapy or in combination with chemotherapy [18]. Therefore, several trials are currently ongoing to delineate new immunotherapy and combinatorial strategies effective for HNSCC patients [19]. Furthermore, the use of immunotherapy in a neoadjuvant setting is particularly attractive. In a TIME previously exposed to therapies and rich of tumor-derived antigens, immunotherapy may indeed enhance the efficacy of standard loco-regional treatments [11].

Overall, new immune-based therapies increasingly rely on an in depth characterization of the tumor-immune cell interactions [20]. Notwithstanding, little is known about the immune contexture diversity between primary tumors and matching metastasis from the same patient. The metastatic lesion hosts cancer cells with metastatic capacity, and thus the biomarker status at the metastatic location might give more relevant prognostic information.

Here, using a combination of immune-related gene expression profiling (GEP), quantitative multiplex immunofluorescence (mIF) and spatial proximity analyses, we provide insights about the TIME characterization of HPV-positive and HPV-negative OPSCC, both on primary and metastatic lesions. We advance a potential new rationale for the incorporation of ICI in the loco-regional therapy strategies for patients with heavily infiltrated treatment-naïve OPSCC, and for the combined use of ICI and tumor-specific T cell response inducers or tumor-associated macrophages (TAM) modulators for the non-inflamed counterparts.

## Methods

### Patients

Thirty-nine consecutive patients undergoing up-front surgery with simultaneous neck dissection for N-positive OPSCC (sub-sites palatine tonsil and base of the tongue) at Treviso Regional Hospital and Trieste University Hospital from August 2010 to January 2021, were included in the study. Patients with previous cancer history or recurrent cancer or distant metastases, and those previously undergoing chemotherapy or radiotherapy were excluded. Clinicopathological information including gender, age, year of diagnosis, cancer sub-site, pTNM stage, histological grading, extracapsular extension, margins status (R), adjuvant treatment, tobacco smoking, alcohol drinking were retrieved from the electronic medical records. Pathologic staging was based on the 8th edition of the American Joint Committee on Cancer (AJCC) staging system. All patients underwent a regular follow-up until death or 31 December 2021. The study was approved by the ethic committees for clinical experimentation of Treviso and Belluno provinces and Friuli Venezia Giulia region, and all patients signed an informed consent.

### HPV analyses and immunohistochemistry for p16^INK4A^ protein expression

Search and typing of HPV DNA sequences were carried out from genomic DNA extracted from formalin-fixed paraffin-embedded (FFPE) sections by QIAamp DNA Mini kit (Qiagen), according to the manufacturer’s instructions. DNA was tested by PCR with MY09/MY11 primers, followed by restriction fragment length polymorphism analysis of the amplification products. The DNA quality of the samples was verified by amplification of the β-globin gene. p16^INK4a^ status was evaluated from FFPE sections by immunohistochemistry using an anti-human p16^INK4a^ antibody (clone G175–405) and the BD Pharmingen™ IHC Detection Kit. p16^INK4a^ positivity was based on a strong and diffuse nuclear and cytoplasmatic staining in at least 70% of tumor cells. A tumor was defined as HPV-positive by double positivity for HPV DNA and p16^INK4a^.

### Immune-related gene expression profiles

Total RNA was extracted from 2 consecutive 10 μm-thick FFPE primary tumor sections, using the RNAesy FFPE kit (Qiagen), according to the manufacturer’s instruction. RNA quantification was performed with Nanodrop 1000 spectrophotometer (ThermoFisher scientific), and the RNA integrity and quality were evaluated with the Agilent 2100 Bioanalyzer System, using the RNA 6000 nano kit (Agilent). The PanCancer Immune Profiling panel (NanoString Technologies) was used to measure the expression of 770 immune-related genes covering innate and adaptive immune responses. The panel included 40 housekeeping genes, 8 negative controls and 6 synthetic positive controls. Samples were processed according to the manufacturer’s instructions provided by NanoString Technologies. RNA (300 ng) from each sample was hybridized with panel probes for 19 hours at 65 °C, and then complexes were processed on the nCounter FLEX platform (NanoString Technologies). Cartridges were scanned at 555 fields of view. Gene expression data were analysed with the nSolver 4.0 Software (NanoString Technologies), and a quality check was performed in the gene expression analysis. Raw data were normalized using a ratio of the expression value to the geometric mean of housekeeping genes on the panel; then, normalized data were Log2 transformed. The nCounter Advanced Analysis module V.2.0.134 software (NanoString Technologies) was used for differential expression analysis and to obtain scores for cell type profiling and pathway analysis, based on the expression of predefined genes (Supplementary Table S1 and S2).

### Multiplex immunofluorescence

mIF staining was performed on primary tumors and lymph node metastases using the Tyramide Signal Amplification (TSA)-based method (Akoya Biosciences), as previously reported [21–24]. Two panels were employed to characterize the subsets of tumor-infiltrating immune cells (Table 1). Sequential 4 μm-thick FFPE tissue sections were deparaffinised in Clearene (Leica Biosystems) and rehydrated by serial passages in graded ethanol. A 20 minutes passage in 10% neutral buffered formalin (Sigma) ensured the fixation of the sample on the glass slide. Heat-induced epitope retrieval (HIER) was performed with a microwave oven using Target Retrieval Solution pH 9 (Dako) or pH 6 (Akoya Biosciences), depending on primary antibody. Tissue sections were blocked with Protein Block Serum-free (Dako) for 10 minutes before applying each primary antibody. The anti-mouse+rabbit Horseradish Peroxidase (HRP)-conjugated secondary antibody (Akoya Biosciences) was added for 10 minutes at room temperature and a different TSA-conjugated Opal fluorophore (Akoya Biosciences) was applied onto the tissues for 10 minutes. Then, the HIER step was performed, and the protocol was repeated sequentially until all markers had been stained. Slides were then counterstained with spectral DAPI (Akoya Biosciences) and mounted using Pro long Diamond anti-fade mounting medium (Invitrogen).

**Table 1:** Multiplexed panels

	Antibody	Clone	Source	Dilution	Opal Fluorophore
**9-color panel**	CD68	KP1	Dako	1:100	Opal-540
	CD8	C8/144B	Dako	1:2500	Opal-620
	FoxP3	D2W8E	Cell Signalling	1:100	Opal-570
	CD163	10D6	Leica Biosystems	1:150	Opal-690
	CD103	EPR4166(2)	Abcam	1:500	Opal-480
	PD-1	EPR4877–2	Abcam	1:100	Opal-520
	PD-L1	E1L3N	Cell Signalling	1:200	Opal-650
	Pan-cytokeratin (CK)	AE1/AE3	Dako	1:300	Opal-780
**6-color panel**	CTLA-4	UMAB249	Biomedical care	1:100	Opal-520
	CD4	4B12	ThermoFisher	1:20	Opal-650
	CD8	C8/144B	Dako	1:2500	Opal-480
	Pan-HLA class I (HLA-I)	EMR8–5	Abcam	1:100	Opal-570
	Pan-cytokeratin (CK)	AE1/AE3	Dako	1:300	Opal-690

### Multispectral imaging, cell density and cell-to-cell distance analyses

At least 20 fields of each multiplex-stained slide were imaged using the Mantra Quantitative Pathology Workstation (Akoya Biosciences) at X20 magnification. For each sample, only areas comprising tumor cells were considered to avoid the acquisition and analysis of normal-like tonsil and lymph node tissues. The inForm Image Analysis software (version 2.4.10, Akoya Biosciences) was used to unmix multispectral images using a spectral library built from acquisition of single fluorophore-stained control tissues, and containing fluorophores-emitting spectral peaks. A selection of representative multispectral images was used to train the inForm software to create algorithms, as previously described [25]. Briefly, tumor tissue was segmented based on recognition of cells staining positive for the pan-cytokeratin antibody, to differentiate infiltrating immune cells within the tumor area and in the surrounding stroma; then, single cells were segmented by nuclear counterstaining. Cell phenotyping was based on the detection of co-localized cell surface or intracellular markers; five algorithms were generated for the 9-color panel, and additional four algorithms for the 6-color panel. The created algorithms were applied in the batch analysis of all acquired multispectral images, and phenoptrReports (add-ins for R Studio from Akoya Biosciences) was used to calculate cell densities, cell percentages and cell-to-cell distances for each sample. For mean distance between different cell subtypes, the nearest neighbors analysis was used, while count within analysis was employed to calculate the percentage of reference cells (tumor or immune cells), among the total number of reference cells, which are present within a 20 μm radius from at least one cell of a different phenotype (Supplementary Fig. S1).

### Statistical analysis

Statistical analyses were carried out using GraphPad Prism software (version 8.0) and IBM SPSS Statistics (version 28). Patient characteristics were evaluated according to the HPV status by using Fisher’s exact test. For continuous variables, median, quartiles and range were described and statistical analyses were performed with the non-parametric two-tailed Mann-Whitney test between two groups. Linear regression analysis was used to investigate differential gene expression using HPV status as a covariate. The Benjamini-Hochberg adjusted *p*-values were used to decrease the false discovery rate. Differentially expressed genes (DEGs) were defined by Log2 fold difference of > 1 or < − 1, and an adjusted *p* value < 0.05. Disease-free survival (DFS) was defined by the time from the last treatment to death for any cause or relapse, whichever occurred first, or to the last follow-up date. A binary value (low vs. high) was assigned to each patient based on the median score, cell density or percentage cut-off for each gene set or marker. The Kaplan-Meier method was used to generate DFS curves and statistical differences were evaluated using the log-rank Mantel-Cox test. Moreover, univariate Cox regression modelling for proportional hazards was used to calculate hazard ratio (HR) and 95% confidence interval (CI) for the association of dichotomized immune variables and patient outcome. For the correlation analyses, the non-parametric Spearman’s correlation coefficient (r) was calculated. All reported *p*-values are two-sided and *p* ≤ 0.05 was considered statistically significant.

## Results

### Patient characteristics

Patient characteristics according to the HPV status are reported in Table 2. Of the 39 patients surgically treated for OPSCC (median age, 61 years), 23 (59.0%) were males and 16 (41.0%) females. The primary tumor was removed using transoral laser microsurgery and open surgery via mandibulotomy or pharyngotomy in 24 and 15 patients, respectively. A clear R0 resection was obtained in all cases. Palatine tonsil was the most frequently involved sub-site (*n* = 32; 82.1%). Twenty-four (61.5%) patients harbored a transforming HPV infection defined by HPV DNA and p16^INK4a^ double positivity, while the remaining 15 cases were double negative. Prevalence of HPV-positive tumors was higher among never smokers. HPV16 was the most prevalent type (*n* = 21; 87.5%), with the remaining subjects being positive for HPV33 (2 cases) and HPV18 (1 case). Twenty-seven/39 patients (69.2%) underwent adjuvant post-operative (chemo)radiation. No significant difference in DFS was observed between HPV-positive and HPV-negative patients (*p* = 0.34).

**Table 2:** Clinical and demographic characteristics of subjects according to HPV status

	All patients		HPV-negative		HPV-positive		
	n	(%)	n	(%)	n	(%)	***p***-value
Age (years)							
Median (range)	61 (51–85)		59 (52–85)		62 (51–85)		.599
Sex							
Woman	16	(41.0)	4	(26.7)	12	(50.0)	.150
Man	23	(59.0)	11	(73.3)	12	(50.0)	
Tobacco smoking							
Never	11	(28.2)	0	(0.0)	11	(45.8)	.020
Ever	28	(71.8)	15	(100.0)	13	(54.02)	
Alcohol drinking							
Never	21	(53.8)	6	(40.0)	15	(62.5)	.141
Ever	18	(46.2)	9	(60.0)	9	(37.5)	
Sub-site							
Base of tongue	7	(17.9)	3	(20.0)	4	(16.7)	.792
Tonsil	32	(82.1)	12	(80.0)	20	(83.3)	
pT^a^							
T1	10	(25.6)	2	(13.3)	8	(33.3)	.146
T2	14	(35.9)	4	(26.7)	10	(41.7)	
T3	9	(23.1)	6	(40.0)	3	(12.5)	
T4	6	(15.4)	3	(20.0)	3	(12.5)	
pN^a^							
N1	21	(53.8)	3	(20.0)	18	(75.0)	.001
N2	14	(35.9)	8	(53.3)	6	(25.0)	
N3	4	(10.3)	4	(26.7)	0	(0.0)	
pStage^a^							
I	13	(33.3)	0	(0.0)	13	(33.3)	<.000
II	10	(25.6)	0	(0.0)	10	(25.6)	
III	3	(7.7)	2	(13.3)	1	(4.2)	
IV	13	(33.3)	13	(86.7)	0	(0.0)	
Grading							
G2	16	(41.0)	9	(60.0)	7	(29.2)	.058
G3	23	(59.0)	6	(40.0)	17	(70.8)	
Adjuvant treatment							
None	12	(30.8)	2	(13.3)	10	(41.7)	.083
(Chemo)-radiotherapy	27	(69.2)	13	(86.7)	14	(58.3)	

^a^ According to TNM classification 8th edition, 2017

### HPV-positive and HPV-negative OPSCC differ in immune signatures

**Fig. 1:**
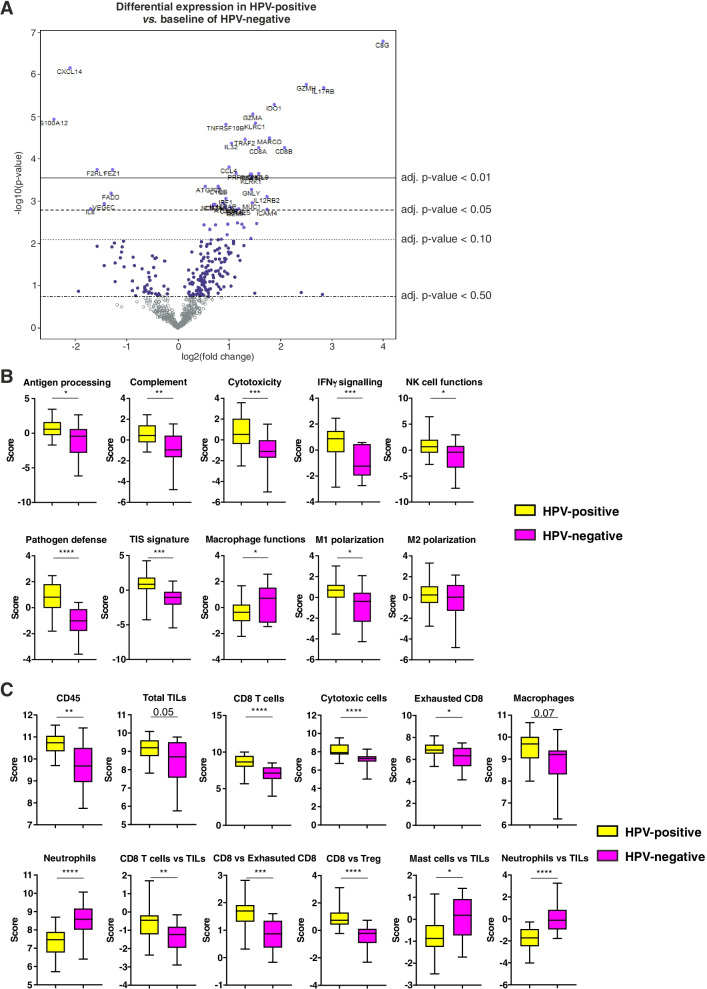
Differential expression of immune-related genes in HPV-positive and HPV-negative OPSCC. (**A**) Volcano plot depicting significantly increased (right) or decreased (left) expression of immune-related genes in HPV-positive OPSCC. (**B**) Differential expression of predefined pathway genes and (**C**) gene expression-based cell types in HPV-positive and HPV-negative OPSCC. Significantly different data are represented by *p < 0.05, **p < 0.01, ***p < 0.001 and ****p < 0.0001

To determine the immune signatures of HPV-positive and HPV-negative OPSCC, we used the NanoString PanCancer Immune Profiling Panel on the entire patient cohort. Gene expression analysis revealed 30 DEGs between HPV-positive and HPV-negative OPSCC (Fig. 1A and Supplementary Table S3). In particular, HPV-positive tumors showed a downregulation of genes associated with neutrophils and their chemotaxis (S100A12, IL8), and an upregulation of genes associated with cytotoxicity (GZMH, GZMA, KLRC1, PRF1, KRLK1, GNLY, GZMK), T cell functions (CD8A, CD8B, IL12RB2, EOMES), macrophages (MARCO, MST1R), and inflammation (C8G, IDO1, IL17RB, IL32, CXCL9, CCL5, CXCR3, ICAM4). Moreover, HPV-positive OPSCC displayed a stronger activation of several immune signalling pathways, including antigen processing, complement, cytotoxicity, IFN-γ signalling, NK cell functions, pathogen defence, tumor-inflammation signature (TIS) and macrophage M1-polarization pathway (Fig. 1B and Supplementary Fig. S2A). Conversely, macrophage functions pathway was the only being downregulated in HPV-positive patients as compared to HPV-negative counterparts (Fig. 1B and Supplementary Fig. S2A). Gene expression-based cell type profiling revealed that HPV-positive OPSCC had an increased infiltration of CD45 cells, and in particular of total tumor infiltrating lymphocytes (TILs), CD8 T cells, cytotoxic cells, exhausted CD8 cells and macrophages, and a decrease in neutrophils infiltration (Fig. 1C and Supplementary Fig. S1B). Furthermore, the ratios between CD8 T cells and TILs, CD8 T cells and exhausted CD8 cells, and CD8 and T regulatory (Treg) cells were higher in HPV-positive as compared to HPV-negative OPSCC (Fig. 1C). Conversely, the ratios between mast cells and TILs and between neutrophils and TILs were lower in HPV-positive tumors (Fig. 1C).

### Immune signatures correlate with DFS in OPSCC

**Fig. 2:**
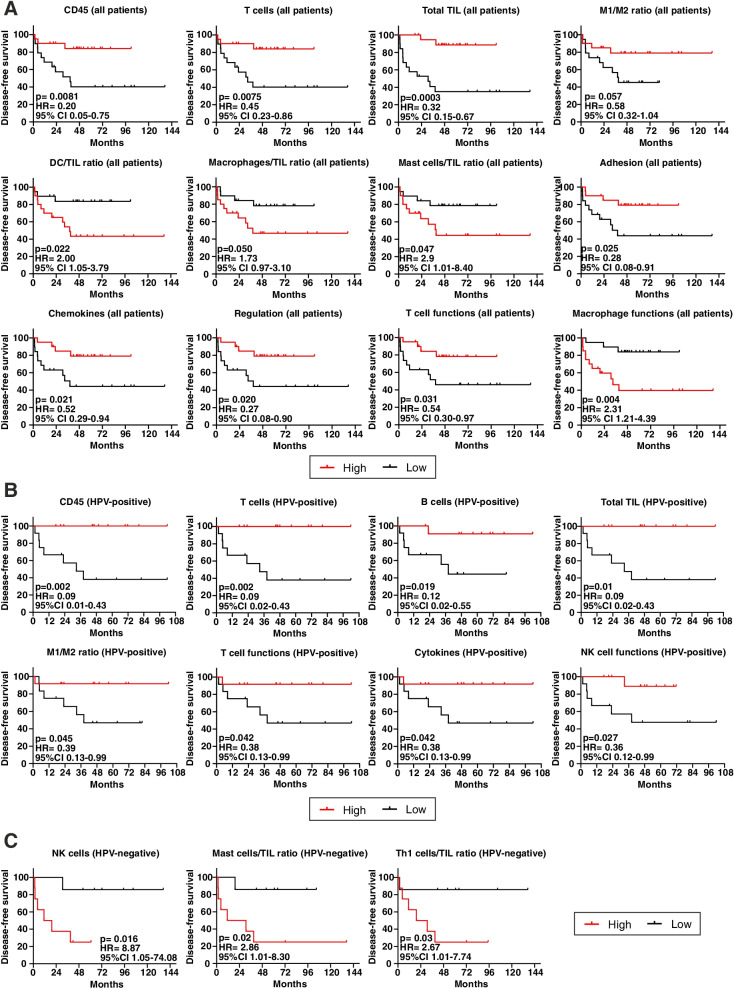
Immune signatures of TIME impact on survival of OPSCC patients. **A-C** Kaplan-Meier survival curves for disease-free survival according to high/low gene expression-based cell types and pathways profiling (classification based on median expression as cut-off), in (**A**) all OPSCC patients (*n* = 39), (**B**) HPV-positive (*n* = 24) and (**C**) HPV-negative patients (*n* = 15). Log-rank statistics were performed to determine significance; p values, hazard ratios (HR) and 95% confidence intervals (CI) are reported in each graph

The assessment of the impact of immune signatures and pathways on DFS in the entire study cohort revealed that patients with a higher expression of CD45, T cells and total TILs signatures, as well as a higher ratio between M1/M2 macrophage signatures, had a longer DFS (Fig. 2A). Conversely, higher ratios between DC and TILs, macrophage and TILs and mast cell and TILs signatures were associated with a shorter DFS (Fig. 2A). Moreover, higher expression of genes associated with adhesion, chemokines, regulation and T cell functions pathways, were prognostic for a better outcome, while the upregulation of macrophage functions pathway was associated with a worse prognosis (Fig. 2A).

Grouping patients according to the HPV status, the higher expression of CD45, T cells, B cells and total TILs signatures were prognostic for a longer DFS in HPV-positive OPSCC, as well as a higher M1/M2 macrophage ratio (Fig. 2B). Furthermore, the upregulation of genes associated with pathways covering T cell functions, cytokines and NK cell functions also correlated with a better prognosis in HPV-positive patients (Fig. 2B). Differently, in HPV-negative patients the higher expression of NK cells gene signatures and the higher ratio between mast cells and TILs or between T helper 1 (Th1) cells and TILs signalling scores, were indicative of a worse prognosis (Fig. 2C).

### HPV-positive primary tumors are heavily infiltrated as compared to HPV-negative counterparts

**Fig. 3:**
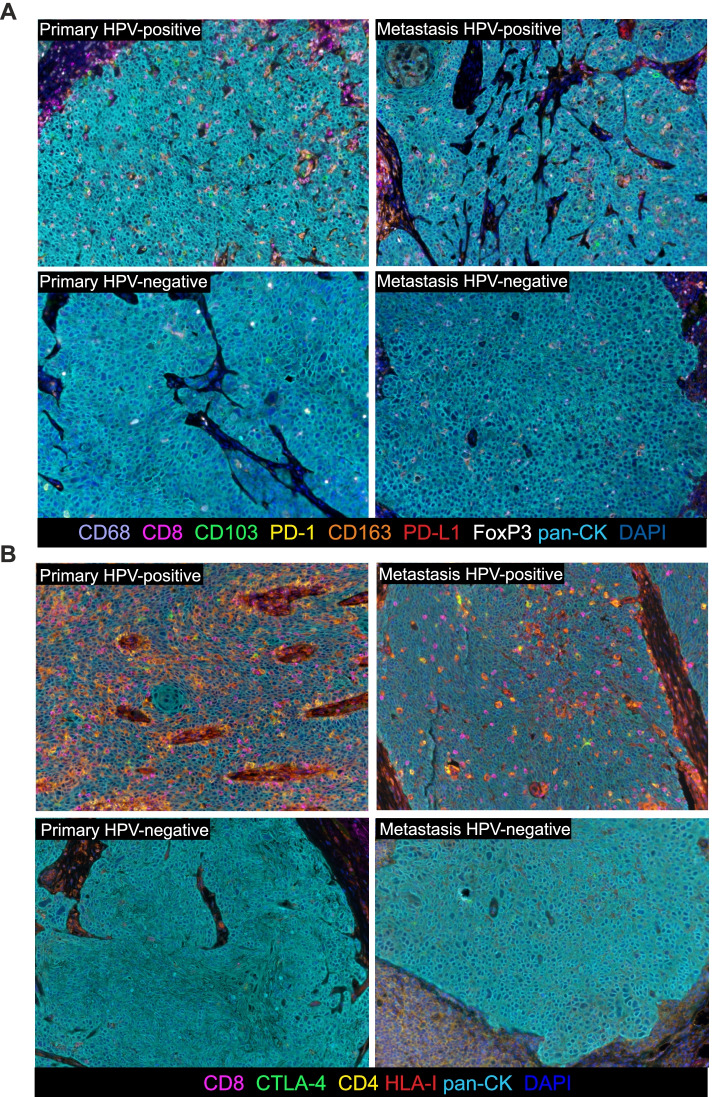
mIF staining of primary HPV-positive and HPV-negative OPSCC and relative lymph node metastasis. (**A**) Representative 9-color and (**B**) 6-color multispectral images at original magnification X20. Immune markers and color codes are indicated in the underlying legend

As recapitulated in the representative Fig. 3, which illustrates the application of the two mIF panels on OPSCC primary tumor and lymph node metastasis sections, it immediately appears evident that HPV-positive lesions are heavily infiltrated as compared to HPV-negative counterparts. The panels included CD8 for CTL, CD68 as a pan-macrophages marker, FoxP3 expressed by Treg, CD163 recapitulating M2-polarized TAM (CD68 + CD163+), CD103 expressed by T_RM_ cells (CD8 + CD103+ cells) and CD4 for T helper lymphocytes. Moreover, the expression of the checkpoint molecules PD-1, PD-L1 and CTLA-4 on T cells, macrophages and tumor cells was also investigated.

**Fig. 4:**
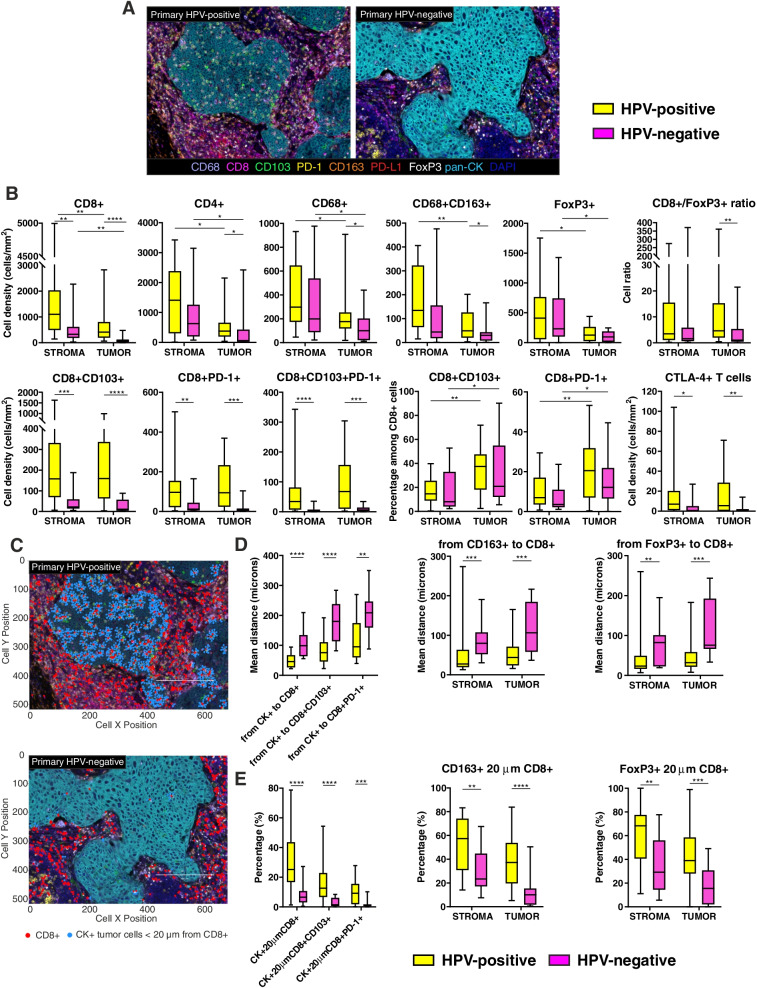
Characterization of immune cells infiltrating HPV-positive and HPV-negative primary tumors. **A** Representative 9-color multispectral images of HPV-positive and HPV-negative primary tumors. Immune markers and color code are indicated in the underlying legend. Original magnification X20. **B** Immune cell populations infiltrating the stromal and the intra-tumoral regions of HPV-positive and HPV-negative primary tumors. **C** Representative images of cell-to-cell distance analysis in HPV-positive and HPV-negative primary tumors. Cancer cells (light blue dots) within a 20 μm radius from CD8+ cells (red dots) are represented. **D** Nearest neighbors analysis measuring the mean distance between each tumor cell and the nearest CD8+, CD8 + CD103+ and CD8 + PD-1+ T lymphocytes (left), or each CD163+ M2-polarized macrophage (middle) or each FoxP3+ Treg cell (right) and the nearest CD8+ T lymphocytes in the stromal and intra-tumoral areas. **E** Count within analysis calculating the percentage of tumor cells within a radius of 20 μm from CD8+, CD8 + CD103+ and CD8 + PD-1+ T cells (left), and the percentage of CD163+ M2-polarized macrophages (middle) or FoxP3+ Treg cells (right) within a radius of 20 μm from CD8+ T lymphocytes in the stromal and intra-tumoral areas. Significantly different data are represented by *p < 0.05, **p < 0.01, ***p < 0.001 and ****p < 0.0001

The density of immune cells infiltrating the microenvironment of OPSCC highly differed according to the HPV status (Fig. 4A). Indeed, in HPV-positive primary tumors we detected a significantly increased intra-tumoral density of CD8+ and CD4+ T lymphocytes, and of CD68+ and CD68 + CD163+ TAMs, as compared to HPV-negative samples, while FoxP3+ Treg cell density was comparable (Fig. 4B). However, the ratio between CD8+ and FoxP3+ cells within the tumor areas was higher in HPV-positive OPSCC (Fig. 4B). The total CD8+ population was further analysed for the co-expression of the integrin CD103, and the checkpoint molecule PD-1. A direct correlation existed between intra-tumoral CD8+ T lymphocytes and double positive CD8 + PD-1+ cells in both groups of patients (*r* = 0.857, *p* < 0.0001 in HPV-positive; *r* = 0.8315, *p* = 0.0002 in HPV-negative). Accordingly, CD8 + CD103+ T_RM_ cells, CD8 + PD-1+ CTL and triple positive CD8 + CD103 + PD-1+ T cell subsets were more represented in HPV-positive than in HPV-negative tumors (Fig. 4B). Of note, although the stromal compartment appeared characterized by higher immune cell densities than the intra-tumoral areas in both patient groups, the percentage of CD8 + CD103+ and CD8 + PD-1+ T cell populations among total CTL were higher within the tumor nests than in the stroma (Fig. 4B). The density of CTLA-4+ T lymphocytes was also investigated, being higher in HPV-positive OPSCC (Fig. 4B).

### In HPV-positive primary tumors, a higher number of tumor cells, M2-polarized macrophages and Treg cells are in closer contact with CD8+ T lymphocytes

Cartographic maps of every acquired field were generated and cell distance analysis was performed (Fig. 4C). As compared to HPV-negative samples, the mean distances observed between tumor cells and CD8+ T lymphocytes, CD8 + CD103+ T_RM_ cells and CD8 + PD-1+ T cells were shorter in HPV-positive primary tumors (Fig. 4D). Moreover, CD8+ T lymphocytes in HPV-positive specimens were closer to M2-polarized TAM and to Treg cells both in the tumor area and in the surrounding stroma (Fig. 4D).

An increased percentage of tumor cells within a 20 μm radius from CD8+ T lymphocytes was observed in HPV-positive OPSCC, as well as from T lymphocytes co-expressing the CD103 or PD-1 molecules (Fig. 4E). Moreover, in HPV-positive tumors almost the 60 and 40% of CD163+ macrophages had CD8+ T lymphocytes within a 20 μm radius in the stroma and within the tumor areas, respectively, as compared to 20 and 10% in HPV-negative OPSCC (Fig. 4E). Furthermore, HPV-positive primary lesions had a higher percentage of FoxP3+ Treg cells within a 20 μm radius from CD8+ CTL, both in the stromal and tumor regions (Fig. 4E).

### A higher immune infiltration characterizes the metastases from HPV-positive patients

**Fig. 5:**
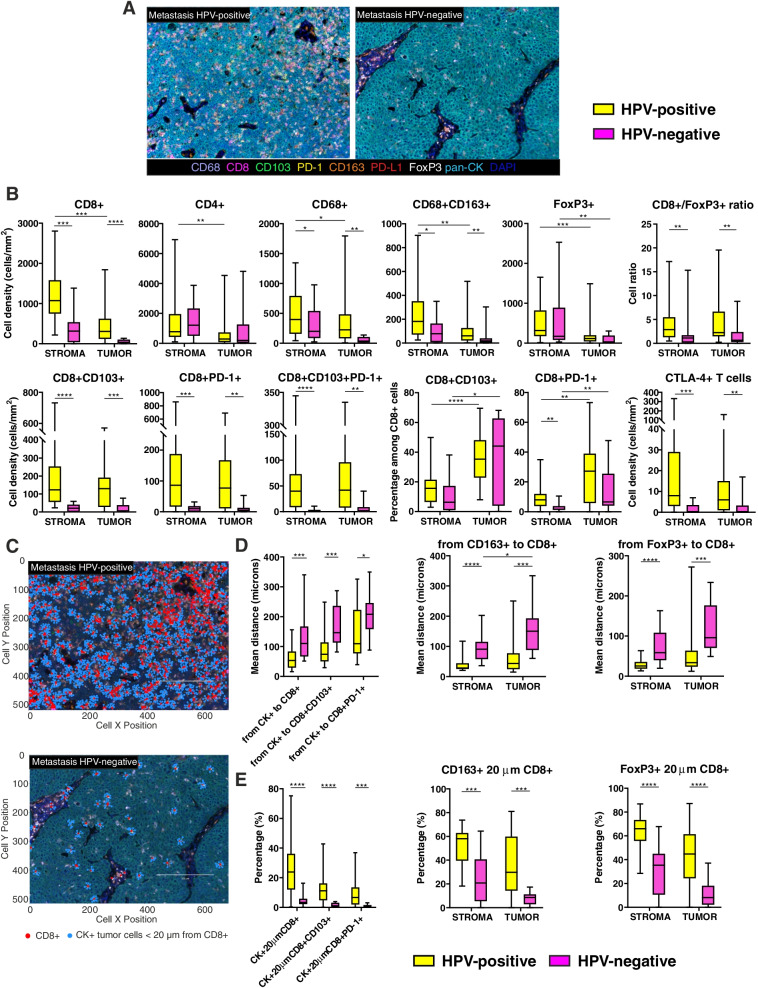
Characterization of immune cells infiltrating HPV-positive and HPV-negative metastases. **A** Representative 9-color multispectral images of HPV-positive and HPV-negative metastases. Immune markers and color code are indicated in the underlying legend. Original magnification X20. **B** Immune cell populations infiltrating the stromal and the intra-tumoral regions of HPV-positive and HPV-negative metastases. **C** Representative images of cell-to-cell distance analysis in HPV-positive and HPV-negative metastases. Cancer cells (light blue dots) within a 20 μm radius from CD8+ cells (red dots) are represented. **D** Nearest neighbors analysis measuring the mean distance between each tumor cell and the nearest CD8+, CD8 + CD103+ and CD8 + PD-1+ T lymphocytes (left), and between each CD163+ M2-polarized macrophage (middle) or each FoxP3+ Treg cell (right) and the nearest CD8+ T lymphocytes in the stromal and intra-tumoral areas. **E** Count within analysis calculating the percentage of tumor cells within a radius of 20 μm from CD8+, CD8 + CD103+ and CD8 + PD-1+ T (left), and the percentage of CD163+ M2-polarized macrophages (middle) or FoxP3+ Treg cells (right) within a radius of 20 μm from CD8+ T lymphocytes in the stromal and intra-tumoral areas. Significantly different data are represented by *p < 0.05, **p < 0.01, ***p < 0.001 and ****p < 0.0001

Metastases from HPV-positive patients were characterized by an overall higher immune cell density than HPV-negative counterparts (Fig. 5A). In particular, an increased density of total CD8+ T lymphocytes, CD68+ macrophages and CD163+ TAMs was observed in the peri-tumoral stroma and within the tumor nests (Fig. 5B). No difference in CD4+ lymphocytes and Treg cell densities existed between the two groups of patients, but the ratio of CD8+ and FoxP3+ cells was higher in HPV-positive metastases (Fig. 5B). A direct correlation between CTL tumor infiltration and PD-1 expression was observed in both patient groups (*r* = 0.7711, *p* < 0.0001 in HPV-positive; *r* = 0.8072, *p* = 0.0008 in HPV-negative). The analysis of CD8+ T cell subsets revealed that CD8 + CD103+, CD8 + PD-1+ and CD8 + CD103 + PD-1+ T lymphocytes were present at higher densities in HPV-positive than HPV-negative metastases, both in the stroma and within the tumor cell nests (Fig. 5B). Whether the percentage of double positive CD8+ populations is calculated among the total CD8+ cells, it becomes evident that their location is prevalently at intra-tumoral level in both patient groups (Fig. 5B). Finally, HPV-positive metastases disclosed a higher density of CTLA-4-expresssing T cells (Fig. 5B).

### In HPV-positive OPSCC lymph node metastases, more tumor and immune regulatory cells are in proximity to CD8+ T lymphocytes

Distance analyses were carried out on lymph node metastases (Fig. 5C), and results resembled those obtained in primary tumors. In HPV-positive metastases, the distance between tumor cells and CTL was shorter than in HPV-negative lesions, regardless the total CD8+ population or the CD103+ or PD-1+ CD8+ subsets (Fig. 5D). Additionally, M2-polarized macrophages and Treg cells mirrored results observed in primary tumors, and appeared closer to CTL in HPV-positive metastases both in the stroma and in the tumor areas (Fig. 5D).

Further, in the microenvironment of HPV-positive OPSCC metastases, CTL established close interactions with the surrounding cancer cells. Indeed, almost 30% of tumor cells in HPV-positive samples had CTL within a 20 μm radius, while this percentage decreased to 4% in HPV-negative lesions (Fig. 5E). Comparable differences were also observed whether considering the proportion of tumor cells within a 20 μm radius from CD8 + CD103+ and CD8 + PD-1+ T lymphocytes (Fig. 5E). Additionally, HPV-positive lymph node metastases revealed higher percentages of CD163+ TAM and FoxP3+ Treg cells detectable within a 20 μm radius from CTL in both stroma and tumor areas (Fig. 5E).

### HPV-positive primary tumors and related metastases have a higher expression of PD-L1

**Fig. 6:**
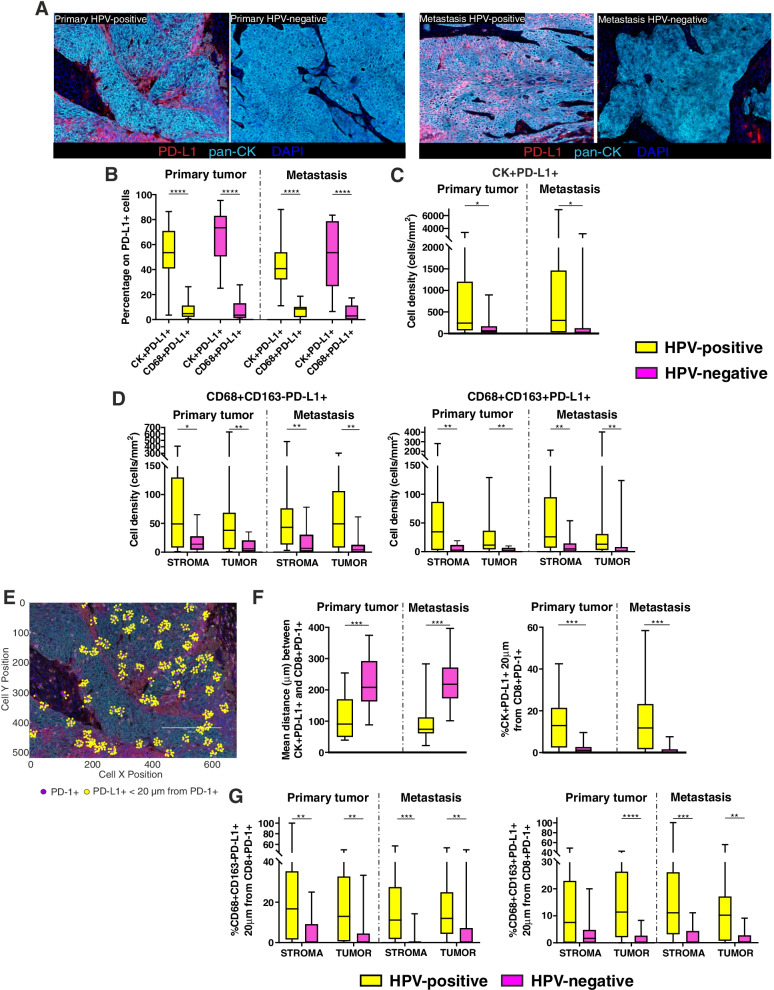
Assessment of PD-L1 expression in HPV-positive and HPV-negative primary tumors and related metastases. **A** Representative multispectral images of HPV-positive and HPV-negative primary tumors (left) and metastases (right). Only PD-L1 (red), pan-cytokeratin (cyan) and DAPI (blue) markers are represented to better appreciate the different PD-L1 expression. Original magnification X20. **B** Assessment of the phenotype of PD-L1+ cells in HPV-positive and HPV-negative primary tumors and metastases. Data are presented as the percentage of PD-L1+ tumor cells (CK+) or macrophages (CD68+) among the total number of PD-L1+ cells. **C** Density of PD-L1+ tumor cells in HPV-positive and HPV-negative primary tumors and metastases. **D** Density of PD-L1+ M1-polarized (CD68 + CD163-) and M2-polarized (CD68 + CD163+) macrophages in the stromal and intra-tumoral areas of HPV-positive and HPV-negative primary tumors and metastases. **E** Representative images of PD-L1+ cells (yellow dots) within a 20 μm radius from PD-1+ cells (purple dots). **F** Mean distance between each PD-L1+ tumor cells and the nearest CD8 + PD-1+ T lymphocytes in HPV-positive and HPV-negative primary tumors and metastases (left); percentage of PD-L1+ tumor cells within a radius of 20 μm from CD8 + PD-1+ T lymphocytes in HPV-positive and HPV-negative primary tumors and metastases (right). **G** Percentage of CD68 + CD163-PD-L1+ macrophages (left) and CD68 + CD163 + PD-L1+ TAM (right) within a radius of 20 μm from CD8 + PD-1+ T lymphocytes in the stromal and intra-tumoral areas of HPV-positive and HPV-negative primary tumors and metastases. Significantly different data are represented by *p < 0.05, **p < 0.01, ***p < 0.001 and ****p < 0.0001

Given the high density of PD-1+ cells in OPSCC samples, we assessed the expression of its ligand PD-L1 that clearly differed between HPV-positive and HPV-negative lesions (Fig. 6A). PD-L1 was mainly expressed by tumor cells in either patient groups (Fig. 6B). However, the density of PD-L1+ tumor cells was higher in HPV-positive than HPV-negative primary lesions and metastases (Fig. 6C). Furthermore, HPV-positive lesions evidenced a higher infiltration of PD-L1+ M1- and M2-polarized macrophages (Fig. 6D). Both in primary tumors and metastases, the increase of macrophage or PD-1+ T_RM_ cell infiltration was associated with increasing PD-L1-expressing macrophages, regardless of HPV status (Supplementary Table S4). Moreover, PD-L1+ cancer cells were directly proportional to PD-L1+ macrophages in HPV-positive and HPV-negative metastatic lesions, and also to CD8 + CD103+ T_RM_ cells in HPV-positive primary tumors (Supplementary Table S4).

### A higher number of PD-L1+ cells are in close contact with PD-1+ T lymphocytes in HPV-positive lesions

To assess whether PD-1+ T cells recruited to the tumor microenvironment were close enough to PD-L1+ tumor cells and macrophages to be potentially affected by this checkpoint axis, we carried out the distance analysis (Fig. 6E). A shorter mean distance between PD-L1+ tumor cells and PD-1+ T cells, as well as a higher percentage of PD-L1+ tumor cells within a 20 μm radius from PD-1+ CTL were found in HPV-positive primary tumors and lymph node metastases, as compared to HPV-negative lesions (Fig. 6F).

Additionally, HPV-positive patients showed a higher percentage of PD-L1+ macrophages having PD-1+ T lymphocytes within a 20 μm radius both in the primary and metastatic lesions (Fig. 6G).

### HLA-I expression on tumor cells varies inter- and intra-individually

**Fig. 7:**
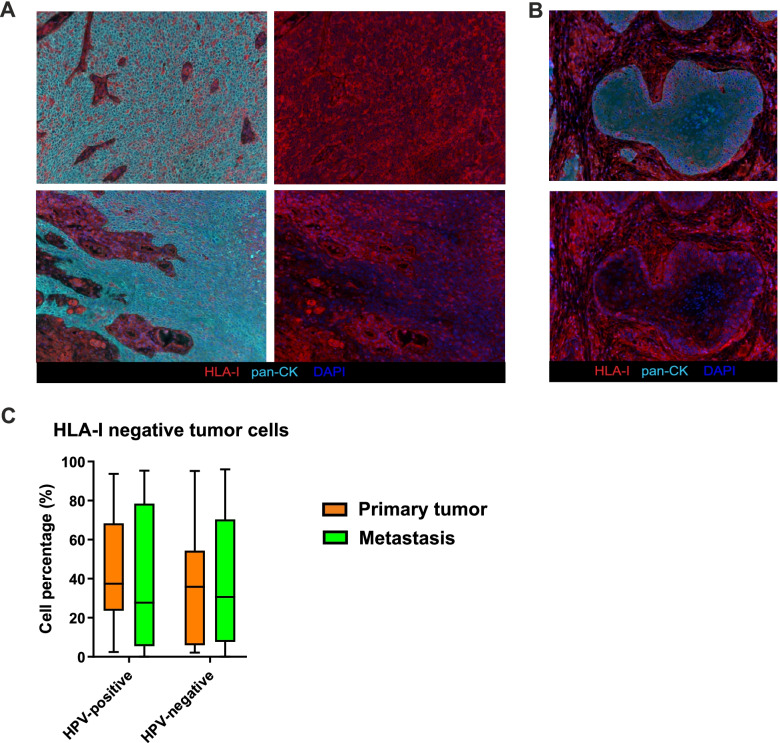
HLA-I expression assessment. **A** Representative multispectral images highlighting the different HLA-I expression on tumor cells in different areas of a tissue section derived from the same patients. In the left pictures, HLA-I (red), pan-CK (cyan) and DAPI (blue) are represented, while in the right pictures the pan-CK channel is switch off to better visualize the HLA-I down-regulation in tumor cells. Original magnification X20. **B** Representative multispectral images which highlight the different HLA-I expression on tumor cells at the tumor-stroma interface. In the left picture, HLA-I (red), pan-CK (cyan) and DAPI (blue) are represented, while in the right picture the pan-CK channel is switch off to better visualize the HLA-I down-regulation in the core of tumor nest. Original magnification X20. **C** Percentage of HLA-I negative tumor cells among the total number of tumor cells in HPV-positive and HPV-negative primary tumors and metastases

Although striking differences in terms of immune infiltrate were detected between HPV-positive and HPV-negative samples, within the primary tumors and the matched lymph node metastases of either groups of patients no significant differences in immune cell densities were observed (Supplementary Fig. S3). Apart the activation status of the infiltrating T cells and the immunomodulatory features of TIME, another escape mechanism adopted by tumor cells to elude CTL recognition and killing is represented by downregulation of the HLA-I molecules [26]. In this regard, assessment of HLA-I on OPSCC neoplastic cells disclosed intra-individual variations, as HLA-I positive areas were frequently flanked by negative tumor nests within the same tissue section (Fig. 7A). Interestingly, HLA-I expression was predominant at the tumor-stroma interface in some samples (Fig. 7B). Additionally, we detected relevant inter-individual variable patterns of expression irrespective of the HPV status, and, as a consequence, the percentage of HLA-I-negative tumor cells among total number of tumor cells did not differ between HPV-positive and HPV-negative lesions (Fig. 7C). Furthermore, an inverse correlation existed between the percentage of tumor cells downregulating HLA-I and the CD8+ T lymphocyte density in HPV-positive primary tumors (*r* = − 0.417, *p* = 0.043).

### The composition of TIME correlates with DFS in OPSCC

**Fig. 8:**
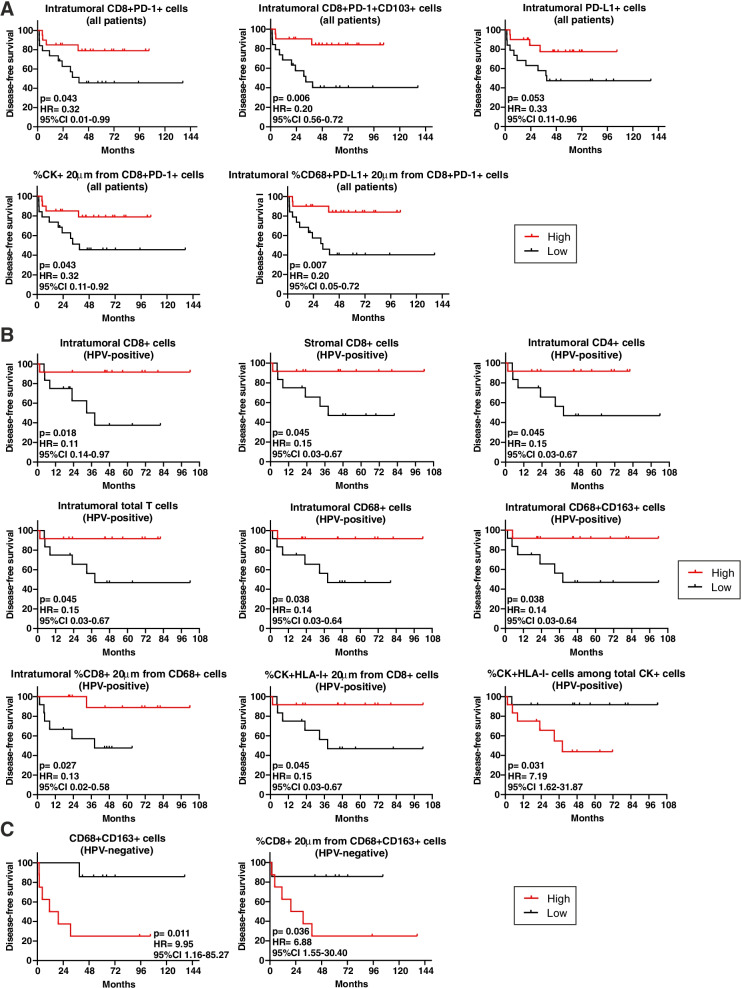
The immune cell composition of the primary tumor microenvironment correlates with patient outcome. **A-C** Kaplan-Meier survival curves for disease-free survival according to the immune cell composition and cell-to-cell interactions of (**A**) all OPSCC (*n* = 39), (**B**) HPV-positive (*n* = 24) and (**C**) HPV-negative (*n* = 15) primary tumors. The median cut-off of each immune cell subset density and percentage was used to separate high and low infiltrated groups. Log-rank *p* values, hazard ratios (HR) and 95% confidence intervals (CI) are reported in each graph

Assessment of the impact of TIME composition and cell-to-cell interactions in primary lesions on DFS in our entire cohort revealed that the higher densities of intra-tumoral CD8 + PD-1+, CD8 + CD103 + PD-1+ and PD-L1+ cells were favorably associated with DFS (Fig. 8A). Moreover, a better clinical outcome was observed in OPSCC patients with a higher frequency of tumor cells or intra-tumoral PD-L1+ macrophages within a 20 μm radius from PD-1+ CTL (Fig. 8A). The positive prognostic role of intra-tumoral T_RM_ cells was maintained even when analysing the metastasis samples (Supplementary Fig. S4).

Stratifying patients according to the HPV status, we assessed that higher densities of stromal or intra-tumoral CD8+ CTL, intra-tumoral CD4+ T cells, total T lymphocytes, CD68+ macrophages and CD163+ TAMs in primary tumors, as well as a higher percentage of CTL in close proximity to macrophages within the tumor nests, were associated with a longer DFS in HPV-positive patients (Fig. 8B). Moreover, HPV-positive patients with a higher amount of HLA-I+ tumor cells close to CD8+ lymphocytes had a better prognosis, while individuals with a higher percentage of tumor cells negative for the HLA-I molecule had a shorter DFS (Fig. 8B). Considering the HPV-positive metastases, the higher densities of CD4+ T cells, intra-tumoral CD8+ T lymphocytes and stromal CD4 + CTLA-4+ T cells were associated with a longer DFS (Supplementary Fig. S4).

Differently from HPV-positive patients, a higher density of M2-polarized TAMs in HPV-negative primary tumors was associated with a shorter DFS (Fig. 8C). Moreover, HPV-negative patients with a higher percentage of CD8+ T lymphocytes within a 20 μm radius from CD163+ macrophages exhibited a worse outcome (Fig. 8C). In HPV-negative metastases, the amount of PD-L1+ tumor cells was favorably associated with prognosis, while patients with a higher density of CTLA-4+ cells in the stroma had a shorter DFS (Supplementary Fig. S4).

### TIME composition of OPSCC exhibits sex-specific differences with distinct prognostic values

**Fig. 9:**
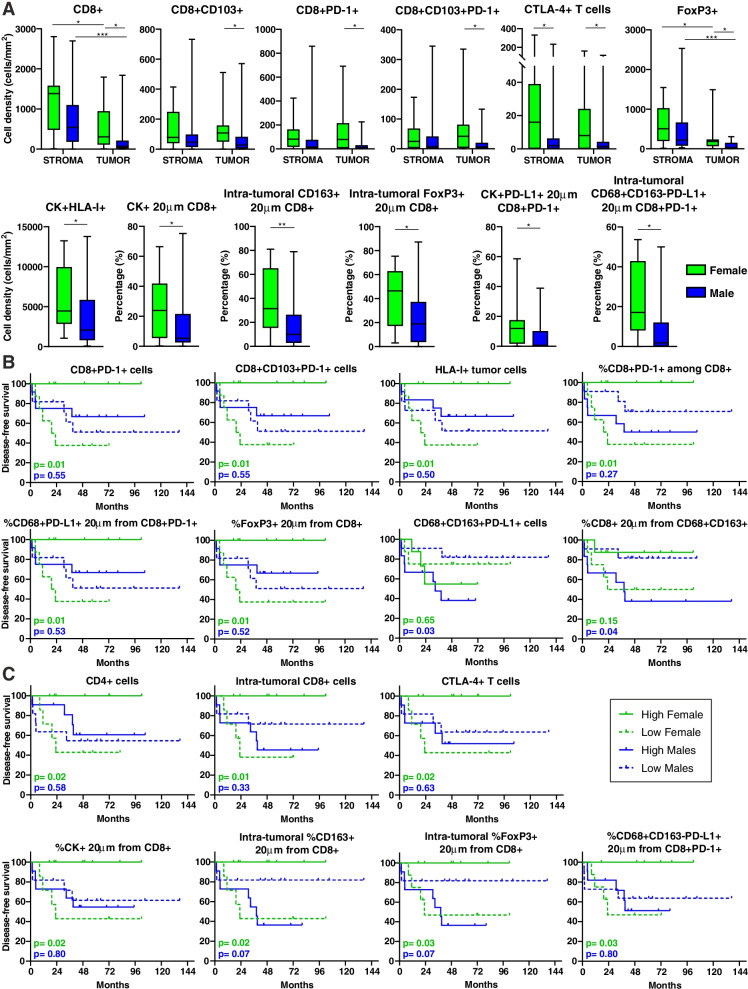
TIME and its prognostic value differ between female and male patients. **A** Density (cells/mm^2^) and count within analyses of immune cell populations infiltrating the stromal and the intra-tumoral regions of lymph node metastases in female and male patients. Significantly different data are represented by *p < 0.05, **p < 0.01 and ***p < 0.001. **B-C** Kaplan-Meier survival curves of disease-free survival for female and male patients according to the immune cell composition and cell-to-cell interactions in (**B**) primary OPSCC and (**C**) lymph node metastases. The median cut-off of each immune cell subset density and percentage was used to separate high and low infiltrated groups. Log-rank p values are reported for each sex in each graph

Patient sex did not impact DFS of our cohort, even considering the overall population (median DFS: 44.8 and 35.6 months in males and females respectively; *p* = 0.72), or stratifying the cohort by HPV status (*p* = 0.21 and *p* = 0.16 in HPV-positive and negative patients, respectively). However, recent evidences highlight the importance of patient sex in anti-tumor immune response, which in turn impinges on the efficacy of ICI [27]. Therefore, we also compared TIME characteristics of males and females. No DEGs were observed considering the entire cohort, as well as grouping patient according to the HPV status. Immune cell populations infiltrating OPSCC primary tumors did not differ between males and females overall considered. Whether patients were stratified according to the HPV status, the only difference observed was a lower density of PD-L1+ macrophages in the stroma of HPV-positive female samples (Supplementary Fig. S5A). On the other hand and regardless the HPV status, several sex-based differences characterized the TIME of lymph node metastases. Indeed, female secondary lesions showed a higher infiltration of CD8+ T lymphocytes within the tumor nests as compared to male samples, and in particular of CD103+ and/or PD-1+ cytotoxic T cells (Fig. 9A). Moreover, female metastases were enriched in CTLA-4+ T cells, intra-tumoral Treg lymphocytes and tumor cells expressing HLA class I, as compared to males (Fig. 9A). Additionally, the percentages of tumor cells, intra-tumoral CD163+ macrophages and Treg cells in close proximity to CD8+ T cells were higher in female metastases than in male lesions, as well as the percentages of PD-L1+ tumor cells and CD163-negative macrophages within a 20 μm radius from PD-1+ CTL (Fig. 9A). Considering HPV-positive metastases, the density of intra-tumoral CTLA-4+ T cells and the percentage of PD-L1+ macrophages interacting with PD-1+ T lymphocytes within the tumor regions were higher in females than in males, while the percentage of HLA-I negative tumor cells was lower (Supplementary Fig. S5B). No differences between men and women were observed in HPV-negative lesions.

To assess whether sex-specific differences of TIME in primary OPSCC and related metastases had a prognostic value, Kaplan-Meier curves stratified by sex were generated for each immune parameter. In primary tumors, a higher density of PD-1+ cytotoxic T lymphocytes, PD-1+ T_RM_ cells and HLA-I+ tumor cells, as well as a higher percentage of PD-1+ CTL among total CD8+ T cells, was associated with a prolonged DFS only in females (Fig. 9B). Moreover, women having a higher percentage of PD-L1+ macrophages in close proximity to PD-1+ CTL, as well as more Treg cells close to CD8+ T lymphocytes, disclosed a better outcome (Fig. 9B). Conversely, a higher density of PD-L1+ M2-polarized macrophages and an increased percentage of CTL within a 20 μm radius from CD163+ macrophages, were associated with a worse outcome in males (Fig. 9B).

In metastases, a higher density of CD4+ T cells, CTLA-4+ T lymphocytes and intra-tumoral CD8+ CTL indicated a better prognosis for female patients (Fig. 9C). Moreover, a higher percentage of tumor cells, intra-tumoral CD163+ macrophages and Treg cells within a 20 μm radius from CTL was associated with a longer DFS only in females, as well as a higher amount of interactions between PD-L1+ macrophages and PD-1+ T cells (Fig. 9C).

## Discussion

PD-1 inhibitors pembrolizumab and nivolumab are approved by the Food and Drug Administration for treatment of recurrent or metastatic HNSCC in the first- and second-line settings [16, 28, 29]. Moreover, ICI therapies are currently being tested in earlier treatment situations, including neoadjuvant setting [30–32]. Unfortunately, only 15–20% of patients with HNSCC benefit from ICI, with this poor outcome being increasingly ascribed to peculiar characteristics of the TIME [33]. Thus, the analysis of the TIME in pre-treatment HPV-positive and HPV-negative patients may critically contribute to rationally identify candidates to immunotherapy.

Here, we report a detailed TIME characterization of treatment-naive HPV-positive and HPV-negative OPSCC primary tumors and matched lymph node metastases, based on multiparametric approaches that define not only the immune gene signatures and the composition of the TIME, but also the cartographic assessment of cell-to-cell interactions. All these data were used to find specific immune signatures, cell populations and spatial interactions capable to stratify OPSCC patients with better outcome.

Previous studies have exploited gene expression analysis to describe the peculiarities of HPV-positive and HPV-negative TIME [34–37]. In our study, the differences in immune gene signatures between the two groups of patients were complemented at a spatial level and validated directly in situ. Overall, markedly increased cytotoxic and immune activation signatures, together with a higher CTL infiltration and closer interactions between CD8+ T lymphocytes and tumor cells, characterize HPV-positive lesions. These results confirm the immune “hot” nature of HPV-positive tumors as compared to the immune “cold” HPV-negative OPSCC microenvironment [38, 39]. Moreover, HPV-positive tumor nests disclosed an increased infiltration by CD8 + CD103+ T_RM_ cells that associate with a better outcome, as previously described [14]. In this regard, T_RM_ lymphocytes have been reported to express molecules involved in cytotoxic activities, such as granzyme B, perforin, IL-2 and IFN-γ, as well as exhaustion markers such as PD-1 and CTLA-4 [40], likely representing tumor-specific effector cells induced by virus antigens [12, 41].

Additionally, the correlation found between the increased tumor-infiltrating CD8+ T cells and PD-1 expression may indicate that the local immune response also induces the PD1/PD-L1 checkpoint pathway, which in turn might limit the capacity of TILs to ultimately eliminate the tumor. Paradoxically but in agreement with Badoual et al. [42], a high density of PD-1+ T lymphocytes in OPSCC primary lesions and their close interactions with cancer cells or PD-L1+ macrophages, is associated with a better prognosis. Since the increase in PD-1 expression may be the result of T cell activation, PD-1 might remain upregulated in the context of a persistent antigen-specific immune stimulation. Furthermore, PD-1+ T cells include potentially tumor-specific T_RM_ cells, exerting anti-neoplastic effects [43]. All these findings support the idea that PD-1 expression should not be merely considered as an exhaustion marker but rather a reflection of the antitumor reactivity, and suggest that patients with high expression of PD-1 on T cells could be potential candidates for anti-PD-1/PD-L1 blockade. Moreover, recent studies demonstrated that the T_RM_ cell subset increases in responder patients with non-small cell lung cancer (NSCLC) and melanoma upon anti-PD-1 administration [44, 45]. Interestingly, data from HNSCC patients treated with neoadjuvant immunotherapy (nivolumab as monotherapy or in combination with ipilimumab) demonstrated that ICI-fostered early intra-tumoral responses are primarily mediated by pre-existing T cell populations with a T_RM_ gene program, which is characterized by tissue residency, cytotoxicity, effector functions and inhibitory receptors including PD-1 [46]. Moreover, authors showed that neoadjuvant ICI can enhance both local and systemic tumor immunity, as they found treatment-induced expansion of emergent T cell clones in tumors and in the peripheral blood, which were undetectable prior to therapy [46]. Overall, our data suggest that neoadjuvant ICI immunotherapy in HNSCC could be regarded as one of the most promising approaches to reactivate and enhance the cytotoxic potential of the tumor-specific T_RM_ cells in “hot” HPV-positive tumors, but also to induce the expansion of anti-tumor T cell clones in “cold” HPV-negative lesions.

More than 50% of HNSCC patients present with metastasis to regional lymph nodes at the time of diagnosis, a feature associated to poor survival and a major prognostic factor for determining the appropriate treatment [47]. Since T cell composition can also vary in the metastatic setting determining different responses to adjuvant therapy, a comprehensive assessment of the TIME in both primary and secondary lesions can provide a more informative view. Irrespective of HPV status and in agreement with previous observations [48, 49], we found that metastases “phenocopied” the originating tumors in terms of immune infiltration and immune checkpoint expression, highlighting the possibility to evaluate the lymph node metastasis specimen if the primary tumor sample is not available for pathological analysis, a not uncommon situation as the presence of neck metastases from hidden HPV-positive OPSCC is a possible clinical manifestation of these malignancies [50].

PD-L1 is considered a predictive marker for response to PD-1/PD-L1 blockade therapies [31]; however, PD-L1 negative tumors sometimes respond to ICI treatment, suggesting the existence of other mechanisms [15]. Differently from Succaria et al. [51], we identified a significantly higher percentage of PD-L1+ cancer cells and macrophages in both HPV-positive primitive and secondary lesions, as compared to HPV-negative samples. In this regard, two mechanisms have been proposed for PD-L1 upregulation [52]. In the innate expression response, PD-L1 upregulation depends on dysregulated oncogenic signalling pathways, and chromosomal alterations and amplifications in the tumor. Thus, PD-L1 expression in cancer cells does not correlate with the nature or the intensity of the local immune response. Conversely, in the adaptive expression response, it is the IFN-γ secreted by activated CTL to induce the upregulation of PD-L1 in tumor cells. Under these latter conditions, therefore, PD-L1 expression is considered a marker of an active host antitumor immune response. Accordingly and regardless the HPV status, in our patient cohort PD-L1 expression was apparently a consequence of an active inflammatory anti-tumor microenvironment involving both myeloid and lymphoid cell populations. Consequently, PD-L1 expression on macrophages and cancer cells was higher in the immune “hot” HPV-positive samples than in the immune “cold” HPV-negative tumors. Our data are consistent with retrospective studies carried out in Merkel cell carcinoma, NSCLC and HNSCC where tumor PD-L1 expression is a positive prognostic factor [53, 54]. That PD-L1 expression is likely an adaptive response in HPV-positive tumors is further supported by the close proximity of PD-L1+ TAM and cancer cells to PD-1+ CTL, which may reflect a potentially active host immunological response otherwise blocked by immune checkpoint interactions. Thus, in a “hot” HNSCC setting, PD-L1 blockade immunotherapy appears particularly promising as a strategy to allow tumor-specific T cells to overcome the shield formed by PD-L1+ tumor cells and to exert their effector activity. Accordingly, an association between PD-1/PD-L1 proximity and better response to anti-PD-1 treatment was reported in Merkel cell carcinoma [55]. On the other hand, these results provide also the rationale to adopt combinatorial therapies [18] to enhance TIL infiltration prior or concurrent with PD-1/PD-L1 blockade immunotherapy, as a good strategy to treat non-inflamed HPV-negative patients expressing low amounts of PD-L1 molecule.

The myeloid cell compartment constitutes another major player in the TIME, and the inflammation associated with tumor and metastasis recruits high amounts of macrophages in the stroma, forming a sort of barrier to obstacle lymphocyte infiltration within the tumor mass [56]. In this regard, evidences in lung squamous-cell carcinoma showed a poor invasion of CD8+ T cells within tumor nests due to long-lasting interactions with TAM in the stroma. Depletion of such TAM restored CD8+ T cell infiltration into tumor islets improving the efficacy of anti-PD-1 immunotherapy [56]. Accordingly, we observed a higher density of macrophages in the stromal compartment and an elevated percentage of CD163+ TAM in close contact with CD8+ T lymphocytes in HPV-positive lesions. However, the prognostic role of such population appears different according to HPV status. Unexpectedly, in HPV-positive lesions the presence of TAMs within the tumor regions and their interactions with CTL has a positive role on patient DFS, probably reflecting an active host anti-tumor immunological response, or a direct role for macrophages in antitumor defence, as already reported in colon cancer [57]. Conversely, higher levels of TAMs and interactions between CD8+ T cells and TAMs negatively associated with the prognosis in HPV-negative patients, in line with previous studies [58, 59]. Taken together, our results highlight the rationale of combining approaches targeting TAMs [60] and immune checkpoint molecules to increase tumor surveillance by CD8+ T cells, and make HNSCC more responsive to anti-PD-1 treatment, particularly in HPV-negative patients.

Sexual dimorphism has been recently ascribed as a relevant factor for cancer incidence and survival [61], even though the role of sex hormones in HNSCC is still controversial and a topic of debate [62]. Evidences highlight the importance of patient sex in modulating the molecular mechanisms that drive the anti-tumor immune response [63]. Accordingly, TIME and levels of immune cell infiltration may differ in males and females with HNSCC, leading in turn to different responses to immunotherapy [64]. In this regard, we found that women had a stronger and more structured immune response in metastatic lesions, as highlighted by the higher abundance of CD8+ T_RM_ cells as well as by the higher percentage of contacts between tumor cells and CTL. On the other hand, OPSCC arising in women apparently develop also complex mechanisms of resistance to counteract such more efficient initial immune recognition and response, as revealed by the higher abundance of Treg cells, the higher expression of multiple checkpoint molecules, and the enrichment of interactions between inhibitory elements and CD8+ lymphocytes. Conversely, we found that the TIME of men secondary lesions was characterized by lower amounts of tumor cells expressing HLA class I, which could lead to a less efficient presentation of tumor neoantigens and potentially explain the poorer immune infiltration. Overall, our results are in line with previous studies performed in different type of malignancies [64–66], and show meaningful sex-based differences in the landscape of OPSCC, as well as in mechanisms exploited by tumors to evade immune response. Importantly, we found remarkable sex-specific differences also in the prognostic value of TIME. As already demonstrated in other tumors [27, 66, 67], our findings suggest that a significant sex-based heterogeneity of response to different type of immunotherapy strategies could be observed in patients with OPSCC, and therefore sex may represent a critical variable in the choice of the optimal treatment for patients with this malignancy.

Finally, some considerations about the limitations of this work. First, there are potential biases due to the retrospective nature of the study, and results must be considered as hypothesis-generating only. Second, the sample size of our cohort is relatively small, and therefore further studies with more OPSCC patients stratified by HPV status are warranted to fully validate our findings. In this regard, however, we want to stress that such patient cohort is quite homogenous in terms of HPV status, sex and anatomical sub-sites of primary and secondary lesions. The third limitation is that the quantity of smoking/drinking is not defined, since we collected data from electronic medical records that usually do not report such data. Fourth, since the main aim of the study was to characterize the TIME both in primary lesions and related lymph node metastases, the present series consists only of patients undergoing upfront surgery that allowed to collect both types of specimens. While this makes the study population more homogeneous, the results relating to the impact of TIME on the outcome can not be extended to patients undergoing upfront (chemo)-radiotherapy. Finally, since only FFPE material was available, the evaluation of the HPV status was based on the double positivity for high-risk HPV-DNA and p16. Although detection of E6 and E7 mRNA would have provided more robust data, double positivity for HPV-DNA and p16 was observed to be the strongest surrogate marker for transforming HPV infection [68, 69].

## Conclusions

Our study clearly establishes that remarkable differences exist in the immune infiltrate between “T-cell-inflamed” HPV-positive and “T-cell-non-inflamed” HPV-negative OPSCC, and that these features are conserved both in primary and metastatic lesions. Moreover, although TAMs and checkpoint molecules expression are generally regarded as immunosuppressive elements, they are not necessarily synonymous of tumor immune evasion and may reflect an ongoing antitumor immune response. Furthermore, we highlight sex-specific differences in the TIME composition of OPSCC and in their prognostic value. Based on these observations, our study provides the rationale for the integration of ICI in the loco-regional therapy strategies for patients with heavily infiltrated treatment-naïve OPSCC, and at the same time advances the notion that approaches combining ICI with tumor-specific T cell response inducers or TAM modulators might be beneficial also for the HPV-negative “cold” counterparts.

## Supplementary Information

**Additional file 1: Supplementary Table S1.** Automatic definition of cell types by NanoString nSolver Software. **Supplementary Table S2.** Automatic definition of immune pathways by NanoString nSolver Software. **Supplementary Fig. S1.** Representative images of cell-to-cell distance analyses. (A) For mean distance between different cell subtypes, the nearest neighbors analysis was used. The mean distance between tumor cells (light blue dots) and the nearest CD8+ cells (red dots) is represented in the figure as an example. (B) The count within analysis was employed to calculate the percentage of reference cells, among the total number of reference cells, which are present within a 20 μm radius from at least one cell of a different phenotype. The percentage of tumor cells (light blue dots) within a 20 μm radius from a CD8+ T lymphocyte (red dots) is represented in the figure as an example. Original magnification X20. **Supplementary Table S3.** Differentially expressed genes (DEGs) between HPV-positive and HPV-negative (used as baseline) OPSCC patients. **Supplementary Fig. S2.** Differential expression of immune-related pathways and cell type genes in HPV-positive and HPV-negative OPSCC. Trend plots depicting differential expression of predefined (A) pathway genes and (B) gene expression-based cell types in HPV-positive and HPV-negative OPSCC. **Supplementary Table S4.** Correlation analysis between immune cell populations in HPV-positive and HPV-negative primary tumors and metastases. **Supplementary Fig. S3.** Immune cells in primary tumors and related metastases. Density (number of cells/mm^2^) of different immune cell populations in HPV-positive and HPV-negative primary tumors and metastases. **Supplementary Fig. S4.** The immune cell contexture of metastases correlates with patient outcome. (A-C) Kaplan-Meier survival curves for disease-free survival according to the immune cell composition of (A) the entire cohort (*n* = 39), (B) HPV-positive (*n* = 24) and (C) HPV-negative (*n* = 15) lymph node metastases. The median cut-off of each immune cell subset density was used to separate high and low infiltrated groups. Log-rank *p* values, hazard ratios (HR) and 95% confidence intervals (CI) are reported in each graph. **Supplementary Fig. S5.** The density of immune cells differs between females and males with HPV-positive lesions. Density (number of cells/mm^2^) of different immune cell populations in females and males with HPV-positive (A) primary tumors and (B) metastases.

## Data Availability

Not applicable.

## Abbreviations

HNSCC: Head and neck squamous cell cancer
OPSCC: Oropharyngeal squamous cell carcinoma
HPV: Human papillomavirus
TIME: Tumor immune microenvironment
CTL: Cytotoxic lymphocytes
IDO-1: Indoleamine 2, 3-dioxygenase 1
T_RM_: Tissue resident memory
ICI: Immune checkpoint inhibitor
PD-1: Programmed cell death protein 1
PD-L1: Programmed cell death protein 1 ligand
GEP: Gene expression profiling
mIF: Multiplex immunofluorescence
TAM: Tumor associated macrophages
pTNM: Pathologic tumor, node, metastasis
AJCC: American Joint Committee on Cancer
FFPE: Formalin fixed paraffine-embedded
PCR: Polymerase chain reaction
TSA: Tyramide signal amplification
HIER: Heat-induced epitope retrieval
HRP: Horseradish peroxidase
CK: Pan-cytokeratin
HLA-I: Human leukocyte antigen class I
DEGs: Differentially expressed genes
DFS: Disease-free survival
HR: Hazard ratio
CI: Confidence interval
IFN-γ: Interferon- γ
NK: Natural killer
TIS: Tumor-inflammation signature
TILs: Tumor infiltrating lymphocytes
Treg: T regulatory cells
Th1: T helper 1
CTLA-4: Cytotoxic T-lymphocyte antigen 4
NSCLC: Non-small cell lung cancer
