# Supplementary material for: The immune microenvironment of HPV-positive and HPV-negative oropharyngeal squamous cell carcinoma: a multiparametric quantitative and spatial analysis unveils a rationale to target treatment-naïve tumors with immune checkpoint inhibitors
Source: J Exp Clin Cancer Res. 2022 Sep 20;41:279. doi: 10.1186/s13046-022-02481-4 (PMC9487049; doi:10.1186/s13046-022-02481-4)
Supplement: Supplementary file 1 — Additional file 1: Supplementary Table S1. Automatic definition of cell types by NanoString nSolver Software. Supplementary Table S2. Automatic definition of immune pathways by NanoString nSolver Software. Supplementary Fig. S1. Representative images of cell-to-cell distance analyses. (A) For mean distance between different cell subtypes, the nearest neighbors analysis was used. The mean distance between tumor cells (light blue dots) and the nearest CD8+ cells (red dots) is represented in the figure as an example. (B) The count within analysis was employed to calculate the percentage of reference cells, among the total number of reference cells, which are present within a 20 μm radius from at least one cell of a different phenotype. The percentage of tumor cells (light blue dots) within a 20 μm radius from a CD8+ T lymphocyte (red dots) is represented in the figure as an example. Original magnification X20. Supplementary Table S3. Differentially expressed genes (DEGs) between HPV-positive and HPV-negative (used as baseline) OPSCC patients. Supplementary Fig. S2. Differential expression of immune-related pathways and cell type genes in HPV-positive and HPV-negative OPSCC. Trend plots depicting differential expression of predefined (A) pathway genes and (B) gene expression-based cell types in HPV-positive and HPV-negative OPSCC. Supplementary Table S4. Correlation analysis between immune cell populations in HPV-positive and HPV-negative primary tumors and metastases. Supplementary Fig. S3. Immune cells in primary tumors and related metastases. Density (number of cells/mm2) of different immune cell populations in HPV-positive and HPV-negative primary tumors and metastases. Supplementary Fig. S4. The immune cell contexture of metastases correlates with patient outcome. (A-C) Kaplan-Meier survival curves for disease-free survival according to the immune cell composition of (A) the entire cohort (n = 39), (B) HPV-positive (n = 24) and (C) HPV-negative (n = 15) lymph node [file 13046_2022_2481_MOESM1_ESM.docx]

**Supplementary Table S1. Automatic definition of cell types by NanoString nSolver Software**

| **Cell Type** | **Gene** | **Cell Type** | **Gene** |
| --- | --- | --- | --- |
| B-Cells | BLK | Macrophages | CD68 |
|  | MS4A1 |  | CD163 |
|  | TNFRSF17 |  | CD84 |
|  | CD19 | Mast Cells | TPSAB1 |
| CD8 T Cells | CD8A |  | MS4A2 |
|  | CD8B | Neutrophils | S100A12 |
| CD45 | PTPRC |  | CSF3R |
| Cytotoxic Cells | GZMB |  | FCGR3A |
|  | PRF1 | NK 56dim Cells | IL21R |
|  | KLRK1 |  | KIR3DL2 |
|  | GZMH |  | KIR3DL1 |
|  | KLRD1 |  | KIR_Inhibiting_Subgroup_2 |
|  | GNLY | NK cells | XCL2 |
|  | GZMA |  | NCR1 |
|  | CTSW | T-Cells | CD3E |
|  | KLRB1 |  | CD3D |
| DC | CD209 |  | CD6 |
|  | HSD11B1 |  | SH2D1A |
|  | CCL13 |  | CD3G |
| Exhausted CD8 | CD244 | Th1 Cells | TBX21 |
|  | EOMES | Treg | FOXP3 |
|  | LAG3 |  |  |

**Supplementary Table S2. Automatic definition of immune pathways by NanoString nSolver Software**

| **Pathway** | **Gene** | **Pathway** | **Gene** | **Pathway** | **Gene** |
| --- | --- | --- | --- | --- | --- |
| Adhesion | ITGA6 | Cell Functions | TP53 | Chemokines | IL13RA2 |
|  | ITGA2 |  | IL13RA2 |  | IRF1 |
|  | ITGB2 |  | IL13RA1 |  | STAT6 |
|  | ITGA4 |  | IRF1 |  | CCL20 |
|  | EPCAM |  | STAT6 |  | IFI27 |
|  | ITGA2B |  | RRAD |  | TNFRSF1B |
|  | CEACAM6 |  | HSD11B1 |  | IL1B |
|  | CEACAM8 |  | PSEN1 |  | IL8 |
|  | ICAM3 |  | FAS |  | LTA |
|  | ITGB3 |  | CD70 |  | TNFRSF1A |
|  | ITGAM |  | TNFSF18 |  | LTBR |
|  | ITGAX |  | LCK |  | IFI16 |
|  | ITGA5 |  | CD5 |  | STAT1 |
|  | MCAM |  | SOCS1 |  | STAT5B |
|  | CEACAM1 |  | IFNG |  | CXCL9 |
|  | ITGAL |  | IL4 |  | CCL5 |
|  | ITGB1 |  | IFNGR1 |  | CXCL3 |
|  | ITGAE |  | IL2 |  | CXCL10 |
|  | ITGB4 |  | IL11 |  | CCR4 |
|  | ICAM2 |  | CR2 |  | STAT3 |
|  | ICAM4 |  | CXCL9 |  | TNFRSF13B |
|  | ITGA1 |  | IRF4 |  | TNFSF4 |
|  | ICAM1 |  | CCR5 |  | KLRB1 |
|  | VCAM1 |  | CXCL10 |  | CXCR1 |
|  | ALCAM |  | IDO1 |  | CCR1 |
| Antigen Processing | THBS1 |  | TBX21 |  | CX3CR1 |
|  | PSMB9 |  | TARP |  | CXCR3 |
|  | HLA-A |  | CCR4 |  | CXCR2 |
|  | CD8A |  | IL5 |  | CCR3 |
|  | TAP1 |  | RORC |  | CCR7 |
|  | HLA-DMB |  | GATA3 |  | CXCR4 |
|  | HLA-DRA |  | CD19 |  | CMKLR1 |
|  | HLA-DRB3 |  | EPCAM |  | XCR1 |
|  | HLA-DRB4 |  | GZMB |  | CXCR6 |
|  | HLA-DPB1 |  | CTLA4 |  | STAT2 |
|  | HLA-DPA1 |  | ICOS |  | IFIT2 |
|  | HLA-C |  | BTLA |  | IFIT1 |
|  | HLA-DQA1 |  | KLRB1 |  | PPBP |
|  | HLA-B |  | KLRC1 |  | CXCL5 |
|  | HLA-DOB |  | ADORA2A |  | IFI35 |
|  | CD1E |  | CCR1 |  | CCL8 |
|  | HLA-DMA |  | CXCR3 |  | CEACAM8 |
|  | PSMB7 |  | CXCR4 |  | IRF8 |
|  | TAP2 |  | PTGDR2 |  | STAT4 |
|  | HLA-DQB1 |  | F2RL1 |  | CCL7 |
|  | MR1 |  | BLK |  | CCL23 |
|  | TAPBP |  | CD3E |  | CX3CL1 |
| B-cell Functions | FAS |  | EOMES |  | IFNAR2 |
|  | CD70 |  | GZMA |  | PSMB8 |
|  | TNFSF18 |  | MS4A1 |  | CSF2RB |
|  | CD5 |  | CD68 |  | IL2RG |
|  | SOCS1 |  | MME |  | CCL21 |
|  | IL11 |  | TNFRSF17 |  | IL6ST |
|  | CR2 |  | CD8A |  | CXCL1 |
|  | IRF4 |  | LAG3 |  | CXCL2 |
|  | CD19 |  | GZMH |  | CCL11 |
|  | CTLA4 |  | LAIR2 |  | CCL18 |
|  | BLK |  | GZMM |  | CCL24 |
|  | CD3E |  | CD1D |  | CCL4 |
|  | MS4A1 |  | STAT4 |  | CCL1 |
|  | CD1D |  | IL18RAP |  | CCL19 |
|  | TNFRSF14 |  | CD8B |  | MS4A2 |
|  | CD80 |  | CD1A |  | CCL22 |
|  | CD86 |  | IL3RA |  | CXCL6 |
|  | CD27 |  | CD7 |  | CCL16 |
|  | CXCR5 |  | DPP4 |  | CCL17 |
|  | ADA |  | FOXP3 |  | CCL26 |
|  | RAG1 |  | IL18 |  | CCL13 |
|  | CD38 |  | IL4R |  | IL22RA1 |
|  | CD79B |  | IL18R1 |  | IL11RA |
|  | CD274 |  | IL21R |  | TNFSF12 |
|  | PTPRC |  | ICOSLG |  | IFNL2 |
| Cell Cycle | THBS1 |  | EGR1 |  | IL32 |
|  | CDKN1A |  | LCP1 |  | CCL15 |
|  | BIRC5 |  | IL12A |  | IL17RB |
|  | BID |  | IL12B |  | A2M |
|  | TNFSF10 |  | NEFL |  | CXCL14 |
|  | ABL1 |  | NRP1 |  | IL17B |
|  | CCND3 |  | TPSAB1 |  | ILF3 |
|  | CXCR4 |  | CD1C |  | CCL25 |
|  | ATM |  | MSR1 |  | CXCR5 |
|  | CASP3 |  | IL13 |  | XCL2 |
|  | BAX |  | TNFRSF14 |  | CCL27 |
|  | BCL2 |  | CD6 |  | C1QBP |
|  | NUP107 |  | KLRD1 |  | CCL14 |
|  | MYD88 |  | AICDA |  | CXCL16 |
| TLR | TLR2 |  | CD209 |  | CXCL11 |
|  | TLR3 |  | CD47 |  | IL19 |
|  | TLR7 |  | CD80 |  | CKLF |
|  | TLR9 |  | IL3 |  | IL22RA2 |
|  | TLR5 |  | IL12RB2 |  | CXCL12 |
|  | TLR4 |  | CD86 |  | CCL2 |
|  | TLR6 |  | KIT |  | CCL3 |
|  | TLR1 |  | CD27 |  | IRF2 |
|  | TLR10 |  | SYT17 |  | CXCL13 |
|  | TLR8 |  | TIGIT |  | TNFSF15 |
| TIS Signature | STAT1 |  | KIR3DL2 |  | CCRL2 |
|  | CXCL9 |  | RPS6 |  | CCL28 |
|  | CCL5 |  | AKT3 | CT Antigen | SEMG1 |
|  | IDO1 |  | F13A1 |  | PRAME |
|  | CMKLR1 |  | CD1B |  | ROPN1 |
|  | CXCR6 |  | KIR3DL3 |  | MAGEA1 |
|  | CD8A |  | KLRC2 |  | MAGEA3 |
|  | LAG3 |  | LILRB1 |  | CTAG1B |
|  | HLA-E |  | KLRF1 |  | MAGEB2 |
|  | CD276 |  | ITGA1 |  | SYCP1 |
|  | PDCD1LG2 |  | LAMP3 |  | CT45A1 |
|  | HLA-DQA1 |  | HAVCR2 |  | MAGEA12 |
|  | CD27 |  | DOCK9 |  | MAGEA4 |
|  | TIGIT |  | GZMK |  | GAGE1 |
|  | PSMB10 |  | CHIT1 |  | MAGEC1 |
|  | CD274 |  | CXCR5 |  | MAGEC2 |
| Complement | C2 |  | CSF3 |  | SPA17 |
|  | C6 |  | KLRK1 |  | DDX43 |
|  | C1R |  | CXCL11 |  | CTAGE1 |
|  | C1S |  | ADA |  | PASD1 |
|  | C1QB |  | RAG1 |  | BAGE |
|  | C8G |  | CD3G |  | PRM1 |
|  | C9 |  | IL17RA |  | SSX1 |
|  | C5 |  | EWSR1 |  | SSX4 |
|  | C8B |  | USP9Y |  | PBK |
|  | C8A |  | SMAD2 |  | SPANXB1 |
|  | C7 |  | PMCH |  | SPACA3 |
|  | C4BPA |  | ANP32B |  | CTCFL |
|  | CCL25 |  | SMPD3 |  | SPO11 |
|  | C1QA |  | LIF | Transporter Functions | FAS |
|  | C4B |  | NCR1 |  | ANXA1 |
| Cytotoxicity | GNLY |  | CD2 |  | TNFSF11 |
|  | HLA-A |  | KLRG1 |  | FYN |
|  | GZMB |  | KIR3DL1 |  | MERTK |
|  | PRF1 |  | KIR_Inhibiting_Subgroup_2 |  | CD36 |
|  | GZMA |  | KIR_Inhibiting_Subgroup_1 |  | CTSW |
|  | GZMH |  | KIR_Activating_Subgroup_1 |  | ITGAM |
|  | GZMM |  | KIR_Activating_Subgroup_2 |  | CD163 |
|  | HLA-C |  | CD38 |  | CRP |
|  | HLA-B |  | TNFSF14 |  | PECAM1 |
|  | GZMK |  | CD79B |  | APOE |
| Leukocyte Functions | IFNG |  | PLA2G6 |  | NT5E |
|  | VEGFA |  | CD274 |  | CD47 |
|  | HCK |  | BATF |  | SIGLEC1 |
|  | SH2D1B |  | MPPED1 |  | ATG7 |
|  | THBD |  | PTPRC |  | CD44 |
|  | CX3CL1 |  | IL12RB1 |  | ATG16L1 |
|  | LCP1 |  | MAF |  | ATG10 |
|  | FUT7 |  | OSM |  | LAMP1 |
| IFN-g Signature | STAT1 |  | LTK |  | FCGR2A |
|  | IFNG |  | REPS1 |  | MFGE8 |
|  | CXCL9 |  | GTF3C1 | Regulation | BCL6 |
|  | CXCL10 |  | FUT5 |  | IRF1 |
|  | IDO1 |  | FEZ1 |  | STAT6 |
|  | HLA-DRA |  | PDGFC |  | THBS1 |
| Cytokines | PTGS2 | Senescence | CDKN1A |  | CDH1 |
|  | IL13RA1 |  | IGF1R |  | RELB |
|  | OAS3 |  | SERPINB2 |  | NFATC2 |
|  | IL1B |  | ABL1 |  | ITGB2 |
|  | IL8 |  | PRKCD |  | CD34 |
|  | CSF2 |  | IRF5 |  | CTSG |
|  | IL1A |  | PLAU |  | LILRB2 |
|  | SPP1 |  | HRAS |  | ELANE |
|  | CD70 |  | EGR1 |  | IL1B |
|  | TNFSF10 |  | FN1 |  | IL8 |
|  | NOD2 |  | ETS1 |  | CSF2 |
|  | IFNG |  | CD44 |  | TCF7 |
|  | LTB | TNF Superfamily | TNFRSF11A |  | FAS |
|  | IL2 |  | TNFRSF1B |  | CD40LG |
|  | IL11 |  | TNFRSF4 |  | CDKN1A |
|  | IL17A |  | TNFRSF11B |  | TGFB1 |
|  | CCL5 |  | FAS |  | BID |
|  | CCR5 |  | TNF |  | CD40 |
|  | VEGFA |  | TNFRSF1A |  | SPN |
|  | CXCL10 |  | CD70 |  | TNFSF10 |
|  | IDO1 |  | TNFRSF9 |  | RUNX3 |
|  | CCR4 |  | TNFSF10 |  | ABL1 |
|  | IL5 |  | TNFRSF10B |  | LCK |
|  | IL7R |  | TNFRSF18 |  | STAT1 |
|  | CCR1 |  | TNFSF18 |  | STAT5B |
|  | JAK3 |  | TNFAIP3 |  | CD5 |
|  | JAK1 |  | TNFSF11 |  | CARD11 |
|  | TYK2 |  | LTB |  | IL4 |
|  | JAK2 |  | TNFRSF13B |  | IL2 |
|  | EBI3 |  | TNFSF4 |  | IL2RA |
|  | CSF3R |  | TNFRSF12A |  | LYN |
|  | IL1R2 |  | TNFRSF17 |  | CXCL9 |
|  | IL1RN |  | TNFRSF8 |  | IRF4 |
|  | FOXP3 |  | TNFRSF13C |  | REL |
|  | IL4R |  | TNFRSF14 |  | IL15 |
|  | IL10RA |  | TNFSF12 |  | CXCL3 |
|  | IL2RB |  | TNFSF13B |  | CXCL10 |
|  | IL6R |  | TNFSF13 |  | TBX21 |
|  | IL12A |  | TNFSF8 |  | CCR4 |
|  | IL12B |  | TNFSF14 |  | IL5 |
|  | IL13 |  | TNFRSF10C |  | STAT3 |
|  | IL23R |  | TNFSF15 |  | SMAD3 |
|  | IL22 | T-cell Functions | TP53 |  | ITGA4 |
|  | IFNL1 |  | IL13RA2 |  | CD19 |
|  | IL12RB2 |  | IL13RA1 |  | HLA-A |
|  | IL26 |  | IRF1 |  | CD160 |
|  | IL21 |  | STAT6 |  | CD247 |
|  | IL9 |  | FAS |  | KLRC1 |
|  | IL5RA |  | CD70 |  | PDCD1 |
|  | HLA-DOB |  | TNFSF18 |  | CXCR1 |
|  | CCL3L1 |  | LCK |  | CCR1 |
|  | IL24 |  | CD5 |  | CXCR2 |
|  | TNFSF8 |  | SOCS1 |  | CCR7 |
|  | CCR2 |  | IFNG |  | CXCR4 |
|  | TNFSF14 |  | IL4 |  | C3AR1 |
|  | FLT3LG |  | IL2 |  | JAK3 |
| Interleukins | IL6 |  | IL11 |  | JAK1 |
|  | IL1B |  | CXCL9 |  | TYK2 |
|  | IL8 |  | IRF4 |  | JAK2 |
|  | IL10 |  | CCR5 |  | STAT2 |
|  | IL1A |  | CXCL10 |  | INPP5D |
|  | TNF |  | IDO1 |  | LAG3 |
|  | TGFB1 |  | TBX21 |  | CCL8 |
|  | IFNG |  | CCR4 |  | EGR2 |
|  | IL4 |  | IL5 |  | ICAM3 |
|  | IL11 |  | CTLA4 |  | IRF8 |
|  | IL17A |  | CCR1 |  | STAT4 |
|  | IL15 |  | CXCR3 |  | CCL23 |
|  | IL5 |  | F2RL1 |  | IFITM1 |
|  | IL17F |  | CD3E |  | CD8B |
|  | IL1RN |  | EOMES |  | CD3D |
|  | IL18 |  | CD8A |  | CD7 |
|  | IL12A |  | LAG3 |  | DPP4 |
|  | IL12B |  | CD1D |  | CD96 |
|  | TGFB2 |  | STAT4 |  | FCGR3A |
|  | IL7 |  | IL18RAP |  | COL3A1 |
|  | IFNA1 |  | CD8B |  | NOTCH1 |
|  | IL13 |  | CD7 |  | RORA |
|  | IL16 |  | DPP4 |  | ICOSLG |
|  | IFNL1 |  | FOXP3 |  | CCL21 |
|  | IL23A |  | IL18 |  | FCGR2B |
|  | IL27 |  | IL4R |  | TAL1 |
|  | IL25 |  | IL18R1 |  | CXCL1 |
|  | IL26 |  | EGR1 |  | CXCL2 |
|  | IL21 |  | LCP1 |  | HMGB1 |
|  | IL32 |  | IL12A |  | IL12A |
|  | IL34 |  | IL12B |  | ITGAL |
|  | IFNA2 |  | CD1C |  | RUNX1 |
|  | IL17B |  | IL13 |  | ITGB1 |
|  | IFNA8 |  | TNFRSF14 |  | FCER1G |
|  | IL19 |  | AICDA |  | TNFRSF13C |
|  | IL24 |  | CD47 |  | CCL24 |
|  | IFNA17 |  | CD80 |  | AMBP |
|  | IFNA7 |  | IL3 |  | C3 |
| Macrophage Functions | CSF2 |  | IL12RB2 |  | TNFRSF14 |
|  | PRKCE |  | CD86 |  | KLRD1 |
|  | IFNG |  | CD27 |  | HLA-E |
|  | SLC11A1 |  | TIGIT |  | HLA-G |
|  | SYK |  | LILRB1 |  | HLA-C |
|  | F2RL1 |  | ITGA1 |  | CCL4 |
|  | DPP4 |  | CXCR5 |  | CD47 |
|  | TICAM1 |  | CXCL11 |  | CD80 |
|  | LCP1 |  | ADA |  | CCL19 |
|  | LBP |  | RAG1 |  | CXCL6 |
|  | CD47 |  | CD3G |  | CCL16 |
|  | CD80 |  | CD2 |  | IL3 |
|  | PSEN2 |  | CD38 |  | NFATC3 |
|  | CD86 |  | TNFSF14 |  | CD276 |
|  | SBNO2 |  | CD274 |  | CD86 |
| NK Cell Functions | IRF1 |  | PTPRC |  | CD200 |
|  | IFNG |  | IL12RB1 |  | CMA1 |
|  | KLRB1 |  | MAF |  | KIR3DL2 |
|  | KLRC1 | M1 polarization | IL6 |  | ICAM2 |
|  | CCR1 |  | TNF |  | CASP3 |
|  | CXCR3 |  | CD40 |  | ICAM4 |
|  | IL18RAP |  | IFNG |  | HLA-B |
|  | CD7 |  | IL2RA |  | SPINK5 |
|  | IL18 |  | IL17A |  | LILRB3 |
|  | IL18R1 |  | CXCL9 |  | KIR3DL3 |
|  | IL12A |  | CXCL10 |  | LILRB1 |
|  | IL12B |  | IL7R |  | ICAM1 |
|  | KLRD1 |  | IL15RA |  | CD3EAP |
|  | IL12RB2 |  | CCR7 |  | HLA-DMA |
|  | KIR3DL2 |  | CD68 |  | SELL |
|  | KIR3DL3 |  | IL12B |  | ULBP2 |
|  | KLRC2 |  | CD80 |  | PVR |
|  | LILRB1 |  | CCL19 |  | BAX |
|  | KLRF1 |  | CD86 |  | SELE |
|  | ITGA1 |  | NOS2A |  | KLRK1 |
|  | KLRK1 |  | CXCL11 |  | LILRA1 |
|  | CXCL11 | M2 polarization | TFRC |  | TNFSF13B |
|  | NCR1 |  | IL10 |  | VCAM1 |
|  | CD2 |  | TGFB1 |  | CD3G |
|  | KLRG1 |  | IL4 |  | MICA |
|  | KIR3DL1 |  | ARG1 |  | CCL3 |
|  | KIR_Inhibiting_Subgroup_2 |  | CXCR4 |  | AMICA1 |
|  | KIR_Inhibiting_Subgroup_1 |  | MS4A1 |  | NFATC1 |
|  | KIR_Activating_Subgroup_1 |  | CD36 |  | TNFSF13 |
|  | KIR_Activating_Subgroup_2 |  | CD163 |  | KLRG1 |
|  | IL12RB1 |  | MSR1 |  | KIR3DL1 |
| Pathogen Defense | OAS3 |  | IL13 |  | KIR_Inhibiting_Subgroup_2 |
|  | CTSG |  | MS4A2 |  | KIR_Inhibiting_Subgroup_1 |
|  | IL1B |  | CCL13 |  | KIR_Activating_Subgroup_1 |
|  | IL8 |  | HAVCR2 |  | KIR_Activating_Subgroup_2 |
|  | CXCL10 |  | MRC1 |  | CD38 |
|  | GNLY |  | CLEC7A |  | TNFSF14 |
|  | PRF1 |  |  |  | IRF2 |
|  | TYK2 |  |  |  | CD81 |
|  | CD8A |  |  |  | PLA2G1B |
|  | IFNAR1 |  |  |  | MICB |
|  | CCL22 |  |  |  | SH2B2 |
|  | PRG2 |  |  |  |  |


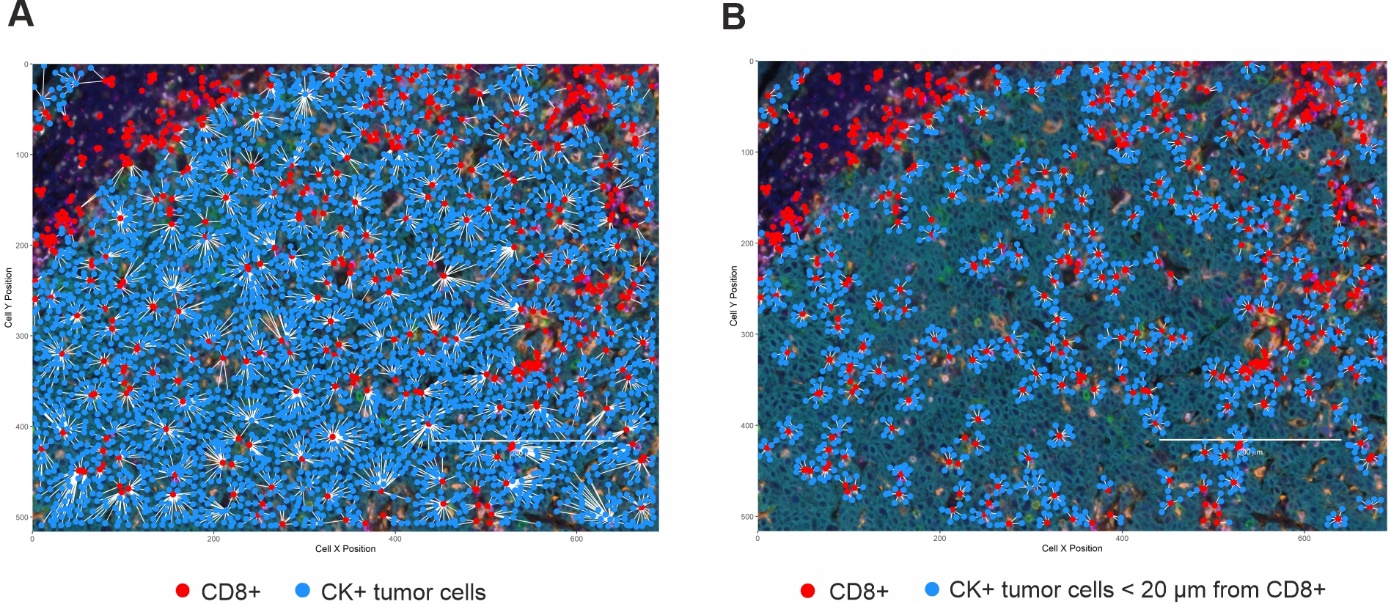


**Supplementary Figure S1. Representative images of cell-to-cell distance analyses**. **(A)** For mean distance between different cell subtypes, the nearest neighbors analysis was used. The mean distance between tumor cells (light blue dots) and the nearest CD8+ cells (red dots) is represented in the figure as an example. **(B)** The count within analysis was employed to calculate the percentage of reference cells, among the total number of reference cells, which are present within a 20 µm radius from at least one cell of a different phenotype. The percentage of tumor cells (light blue dots) within a 20 μm radius from a CD8+ T lymphocyte (red dots) is represented in the figure as an example. Original magnification X20.

**Supplementary Table S3. Differentially expressed genes (DEGs) between HPV-positive and HPV-negative (used as baseline) OPSCC patients**

| **DEGs** | **Log2 fold change** | **BH.p.value** | **Gene.sets** |
| --- | --- | --- | --- |
| C8G | 3.99 | 0.000105 | Complement |
| CXCL14 | -2.1 | 0.000222 | Chemokines |
| GZMH | 2.5 | 0.000326 | Cell Functions, Cytotoxicity |
| IL17RB | 2.84 | 0.000326 | Chemokines |
| IDO1 | 1.88 | 0.000649 | Cytokines, IFN-γ, T-Cell Functions, Tumor-Inflammation Signature |
| GZMA | 1.46 | 0.000925 | Cell Functions, Cytotoxicity |
| S100A12 | -2.42 | 0.00104 | Inflammation, Chemotaxis |
| KLRC1 | 1.51 | 0.00106 | NK Cell Functions, Regulation |
| MARCO | 1.79 | 0.00196 | Macrophage Functions |
| TRAF2 | 1.31 | 0.00196 | TNF signalling |
| IL32 | 1.05 | 0.00225 | Chemokines, Interleukins |
| CD8A | 1.58 | 0.00242 | Antigen Processing, Pathogen Defense, T-Cell Functions, Tumor-Inflammation Signature |
| CD8B | 2.08 | 0.00242 | Regulation, T-Cell Functions |
| F2RL1 | -1.57 | 0.00661 | Macrophage Functions, T-Cell Functions |
| FEZ1 | -1.27 | 0.00661 | Cell Functions |
| PRF1 | 1.13 | 0.00693 | Cytotoxicity, Pathogen Defense |
| CXCL9 | 1.58 | 0.00693 | Chemokines, IFN-γ, Regulation, T-Cell Functions, Tumor-Inflammation Signature |
| CCL5 | 1.41 | 0.00693 | Chemokines, Cytokines, Tumor-Inflammation Signature |
| MST1R | 1.44 | 0.00693 | Macrophage Functions |
| KLRK1 | 1.43 | 0.00806 | NK Cell Functions, Regulation |
| GNLY | 1.43 | 0.013 | Cytotoxicity, Pathogen Defense |
| FADD | -1.3 | 0.0152 | Apoptosis |
| IL12RB2 | 1.74 | 0.0176 | Cytokines, NK Cell Functions, T-Cell Functions |
| MUC1 | 1.45 | 0.022 | Cell Functions |
| VEGFC | -1.44 | 0.022 | Angiogenesis |
| CXCR3 | 1.03 | 0.0246 | Chemokines, NK Cell Functions, T-Cell Functions |
| IL8 | -1.7 | 0.0253 | Chemokines, Cytokines, Interleukins, Pathogen Defense, Regulation |
| EOMES | 1.19 | 0.0253 | T-Cell Functions |
| ICAM4 | 1.74 | 0.0257 | Adhesion, Regulation |
| GZMK | 1.14 | 0.0257 | Cell Functions, Cytotoxicity |


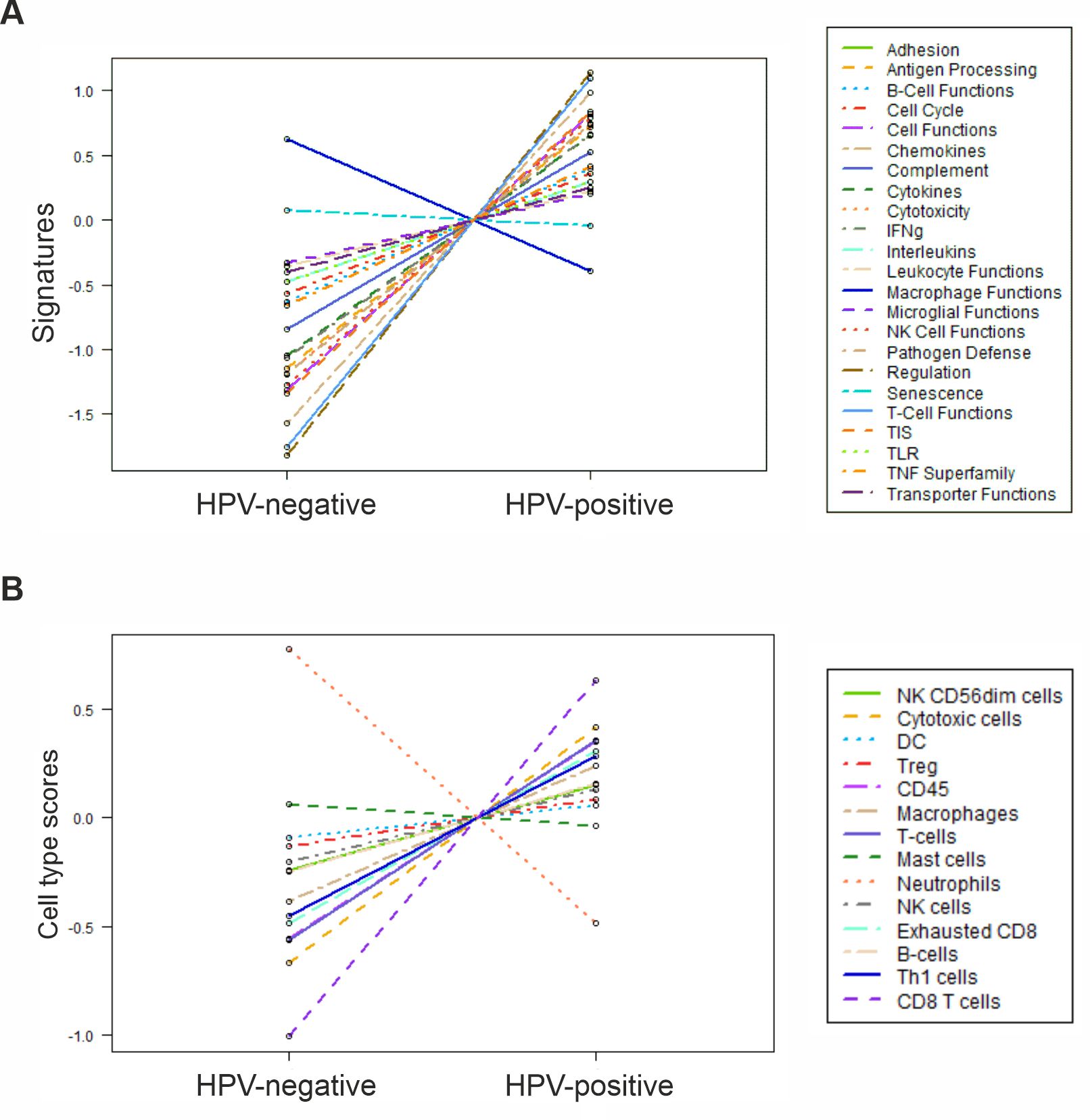


**Supplementary Figure S2. Differential expression of immune-related pathways and cell type genes in HPV-positive and HPV-negative OPSCC.** Trend plots depicting differential expression of predefined **(A)** pathway genes and **(B)** gene expression-based cell types in HPV-positive and HPV-negative OPSCC.

**Supplementary Table S4. Correlation analysis between immune cell populations in HPV-positive and HPV-negative primary tumors and metastases.**

|  |  | **Spearman's Rho** | **Significance**  **(2-tailed)** | **95% Confidence Intervals** | |
| --- | --- | --- | --- | --- | --- |
|  |  |  |  | **Inferior** | **Superior** |
| **HPV-positive primary tumors** | CD68+ vs CD68+PD-L1+ | 0.686 | 0.000 | 0.380 | 0.857 |
|  | CD68+ vs CD68+CD163+PD-L1+ | 0.630 | 0.001 | 0.292 | 0.828 |
|  | CD68+CD163+ vs CD68+PD-L1+ | 0.578 | 0.003 | 0.216 | 0.801 |
|  | CD68+CD163+ vs CD68+CD163+PD-L1+ | 0.634 | 0.001 | 0.298 | 0.830 |
|  | CD8+CD103+ vs CD68+PD-L1+ | 0.477 | 0.019 | 0.078 | 0.744 |
|  | CD8+CD103+PD-1+ vs CD68+PD-L1+ | 0.410 | 0.047 | -0.005 | 0.704 |
|  | CK+PD-L1+ vs CD8+CD103+ | 0.467 | 0.021 | 0.066 | 0.738 |
|  | CK+PD-L1+ vs CD68+CD163+ | 0.487 | 0.016 | 0.091 | 0.750 |
|  | CK+PD-L1+ vs CD68+PD-L1+ | 0.736 | 0.000 | 0.463 | 0.881 |
|  | CK+PD-L1+ vs CD68+CD163+PD-L1+ | 0.743 | 0.000 | 0.475 | 0.885 |
|  | CD68+ vs %CK+PD-L1+ among CK+ | 0.460 | 0.024 | 0.057 | 0.734 |
|  | CD68+PD-L1+ vs %CK+PD-L1+ among CK+ | 0.750 | 0.000 | 0.487 | 0.888 |
|  | CD68+CD163+PD-L1+ vs %CK+PD-L1+ among CK+ | 0.654 | 0.001 | 0.330 | 0.841 |
| **HPV-negative primary tumors** | CD68+ vs CD68+PD-L1+ | 0.804 | 0.000 | 0.482 | 0.934 |
|  | CD68+ vs CD68+CD163+PD-L1+ | 0.732 | 0.002 | 0.337 | 0.908 |
|  | CD68+CD163+ vs CD68+PD-L1+ | 0.618 | 0.014 | 0.138 | 0.863 |
|  | CD68+CD163+ vs CD68+CD163+PD-L1+ | 0.868 | 0.000 | 0.630 | 0.957 |
|  | CD8+ vs CD68+PD-L1+ | 0.514 | 0.050 | -0.014 | 0.818 |
|  | CD8+CD103+ vs CD68+PD-L1+ | 0.614 | 0.015 | 0.132 | 0.861 |
|  | CD8+CD103+PD-1+ vs CD68+PD-L1+ | 0.514 | 0.050 | -0.014 | 0.818 |
|  | CD8+PD-1+ vs CD68+PD-L1+ | 0.714 | 0.003 | 0.303 | 0.901 |
| **HPV-positive metastases** | CD68+ vs CD68+PD-L1+ | 0.881 | 0.000 | 0.731 | 0.950 |
|  | CD68+ vs CD68+CD163+PD-L1+ | 0.871 | 0.000 | 0.708 | 0.945 |
|  | CD68+CD163+ vs CD68+PD-L1+ | 0.684 | 0.000 | 0.367 | 0.858 |
|  | CD68+CD163+ vs CD68+CD163+PD-L1+ | 0.775 | 0.000 | 0.523 | 0.902 |
|  | CD8+ vs CD68+PD-L1+ | 0.484 | 0.019 | 0.077 | 0.753 |
|  | CD8+ vs CD68+CD163+PD-L1+ | 0.422 | 0.045 | -0.001 | 0.717 |
|  | CD8+CD103+ vs CD68+PD-L1+ | 0.432 | 0.039 | 0.012 | 0.723 |
|  | CD8+CD103+ vs CD68+CD163+PD-L1+ | 0.437 | 0.037 | 0.017 | 0.726 |
|  | CD8+CD103+PD-1+ vs CD68+PD-L1+ | 0.476 | 0.022 | 0.066 | 0.748 |
|  | CD8+CD103+PD-1+ vs CD68+CD163+PD-L1+ | 0.526 | 0.010 | 0.133 | 0.776 |
|  | CK+PD-L1+ vs CD68+CD163+ | 0.619 | 0.002 | 0.266 | 0.826 |
|  | CK+PD-L1+ vs CD68+PD-L1+ | 0.632 | 0.001 | 0.286 | 0.833 |
|  | CK+PD-L1+ vs CD68+CD163+PD-L1+ | 0.590 | 0.003 | 0.223 | 0.811 |
|  | CD68+ vs %CK+PD-L1+ among CK+ | 0.448 | 0.032 | 0.031 | 0.732 |
|  | CD68+PD-L1+ vs %CK+PD-L1+ among CK+ | 0.668 | 0.000 | 0.342 | 0.851 |
|  | CD68+CD163+PD-L1+ vs %CK+PD-L1+ among CK+ | 0.629 | 0.001 | 0.281 | 0.831 |
| **HPV-negative metastases** | CD68+ vs CD68+PD-L1+ | 0.714 | 0.004 | 0.280 | 0.906 |
|  | CD68+CD163+ vs CD68+PD-L1+ | 0.688 | 0.007 | 0.231 | 0.896 |
|  | CD8+ vs CD68+PD-L1+ | 0.648 | 0.012 | 0.163 | 0.881 |
|  | CD8+CD103+ vs CD68+PD-L1+ | 0.616 | 0.019 | 0.110 | 0.868 |
|  | CD8+CD103+PD-1+ vs CD68+PD-L1+ | 0.629 | 0.016 | 0.131 | 0.874 |
|  | CD8+PD-1+ vs CD68+PD-L1+ | 0.568 | 0.034 | 0.036 | 0.849 |
|  | CK+PD-L1+ vs CD68+CD163+PD-L1+ | 0.639 | 0.014 | 0.146 | 0.877 |


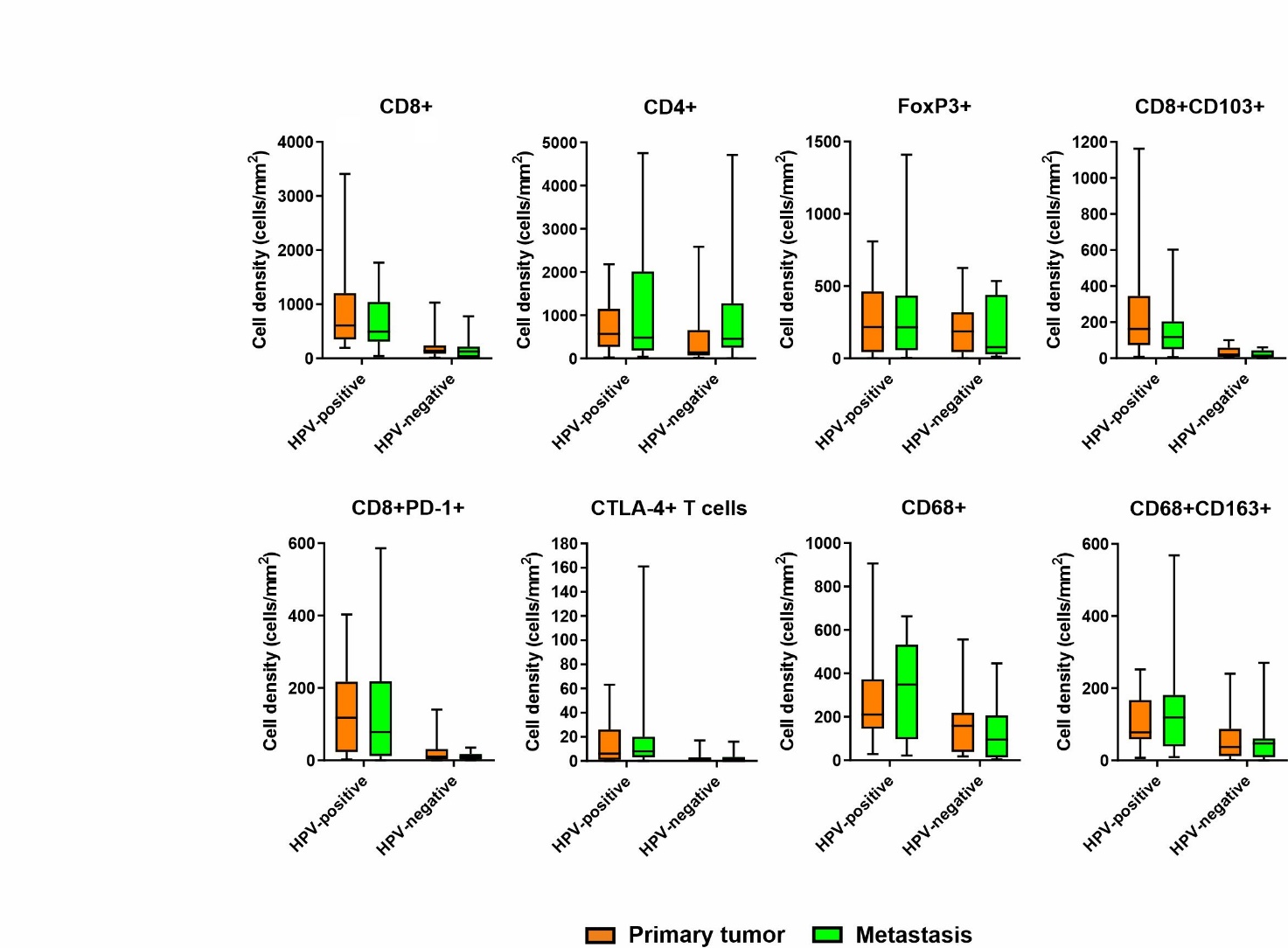


**Supplementary Figure S3. Immune cells in primary tumors and related metastases.** Density (number of cells/mm^2^) of different immune cell populations in HPV-positive and HPV-negative primary tumors and metastases.


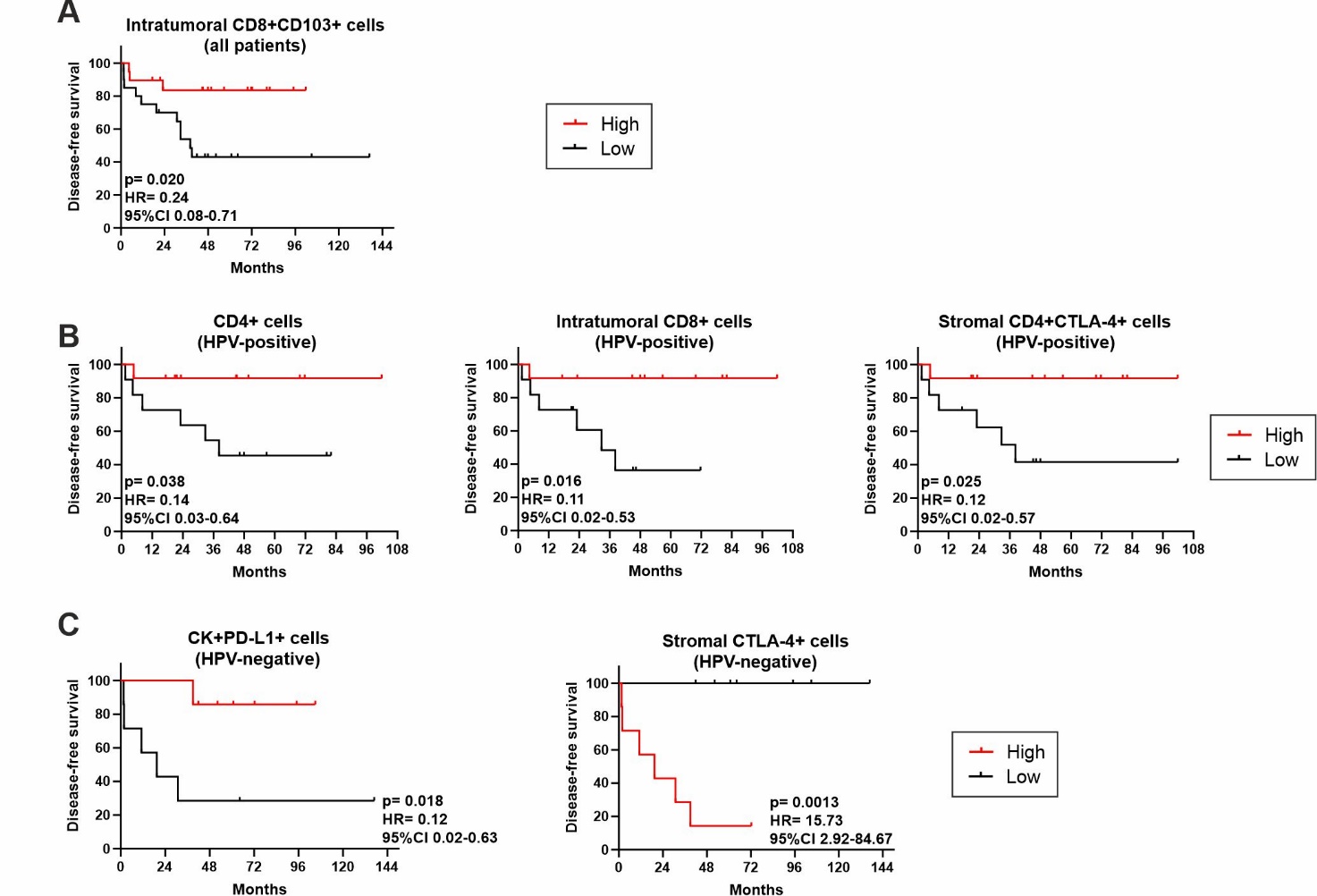


**Supplementary Figure S4.** **The immune cell contexture of metastases correlates with patient outcome.**

**(A-C)** Kaplan-Meier survival curves for disease-free survival according to the immune cell composition of **(A)** the entire cohort (n=39), **(B)** HPV-positive (n=24) and **(C)** HPV-negative (n=15) lymph node metastases. The median cut-off of each immune cell subset density was used to separate high and low infiltrated groups. Log-rank p values, hazard ratios (HR) and 95% confidence intervals (CI) are reported in each graph.


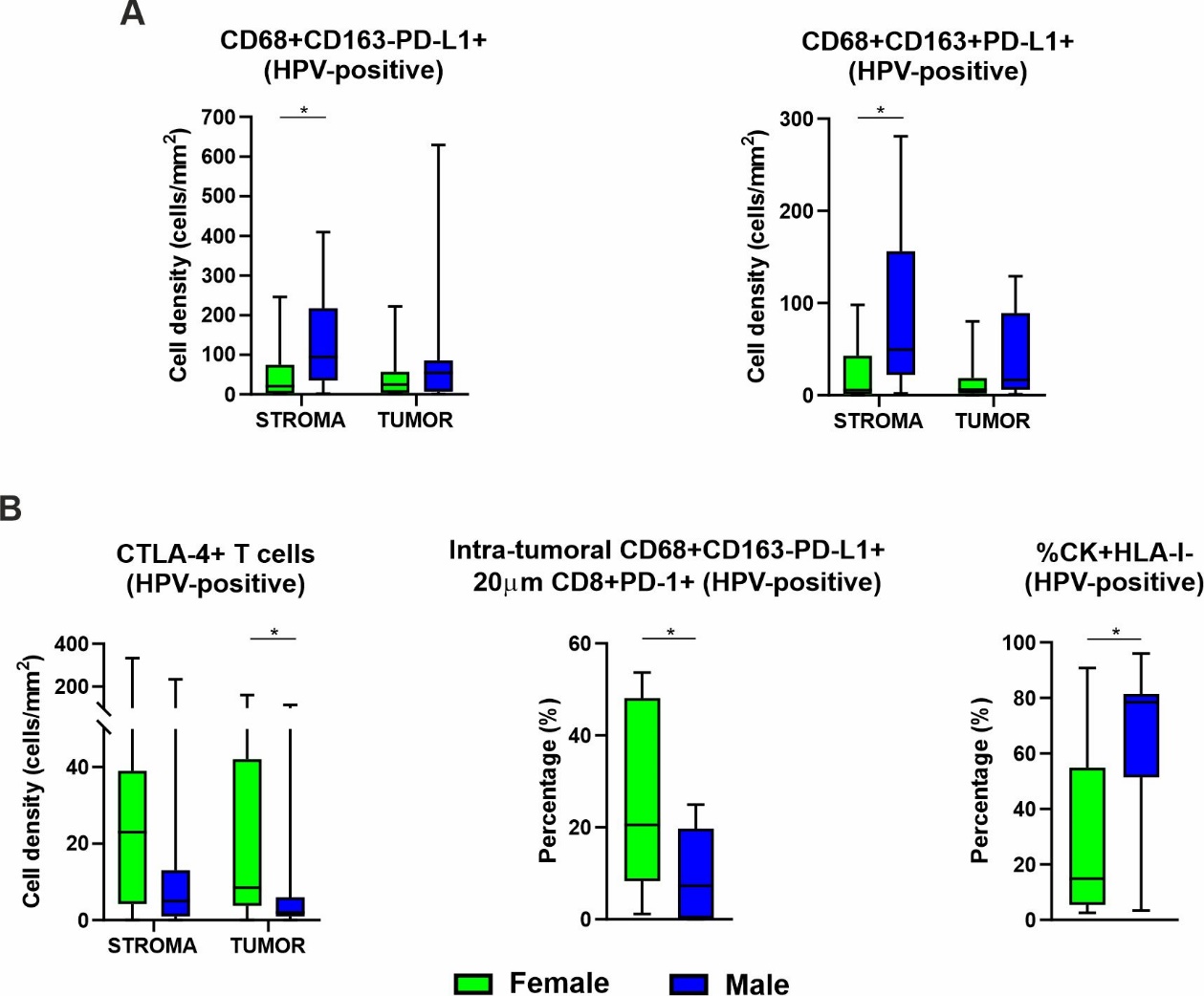


**Supplementary Figure S5. The density of immune cells differs between females and males with HPV-positive lesions.** Density (number of cells/mm^2^) of different immune cell populations in females and males with HPV-positive **(A)** primary tumors and **(B)** metastases.
